# Maximizing mobility: Distributed controllers of a Segway swarm for autonomous navigation in smart city environments

**DOI:** 10.1371/journal.pone.0356369

**Published:** 2026-08-28

**Authors:** Ronal Pranil Chand, Ravinesh Chand, Kritika Lal, Bibhya Sharma, Sandeep Ameet Kumar

**Affiliations:** 1 School of Mathematical & Computing Sciences, Fiji National University, Suva, Fiji; 2 School of Information Technology, Engineering, Mathematics & Physics, The University of the South Pacific, Suva, Fiji; Qingdao University, CHINA

## Abstract

The advancement of autonomous multi-robot systems is critical for addressing the mobility challenges within modern smart city environments. Distributed, acceleration-based controllers are designed and mathematically validated for a Segway swarm navigating cluttered, time-varying urban settings. A Lyapunov-based Control Scheme (LbCS) is utilized to develop a potential function governing collective swarm behavior. This function ensures target convergence, enforces formation cohesion, and prevents inter-agent collisions. It also dictates safe navigation around static geometric constraints (modeled as disks and lines) and the evasion of independent dynamic obstacles. System stability is formally proven, demonstrating asymptotic controllability and convergence to the desired equilibrium state. Numerical simulations verify the framework’s performance across four distinct scenarios, highlighting emergent reactive behaviors to moving entities. These results establish control strategies for the safe collective operation of non-holonomic robots, providing a theoretical foundation for future prototype development in smart city applications.

## 1 Introduction

The continuous expansion of modern infrastructure, particularly within the Smart City framework, necessitates highly adaptable micromobility solutions [1]. Global urbanization is steadily increasing, and projections indicate that over 68% of the world’s population will reside in urban areas by 2050. Consequently, conventional centralized transportation networks face severe scalability limits [2]. The current urban transport model exhibits a distinct mobility gap, characterized by the inefficiency of using large, heavy vehicles for short-distance individual transit [3,4]. To resolve these limitations, robotics research has increasingly shifted toward multi-robot systems. Deploying autonomous fleets rather than individual units offers substantial operational advantages, including expanded coverage, system redundancy, and parallel task execution [5]. The coordination of these fleets draws heavily from biological models. Swarm Intelligence, originating from the study of social insect behaviors red (e.g., ants and bees), describes how simple agents interacting through local rules can generate complex, coherent group behaviors without a central commanding unit [5,6]. Swarm Robotics translates these biological principles to artificial agents, creating decentralized systems capable of adapting to changing environments and persisting despite individual unit malfunctions [7].

Broader advancements in robotic control provide essential methodologies for these multi-agent systems. Recent research spans diverse applications, including the design of specialized robotic manipulators for the automotive industry [8] and exact trajectory tracking solutions for industrial robots [9]. Mobile platform navigation has also advanced through the integration of inertial navigation systems [10] and machine vision for complex motion measurement [11]. Importantly, sophisticated collision detection algorithms [12] establish the foundational safety protocols required for dynamic environments. Building upon these cross-disciplinary methods, the present study translates advanced trajectory generation and collision avoidance strategies to the specific kinematic challenges of under-actuated Segway swarms.

**Fig 1:**
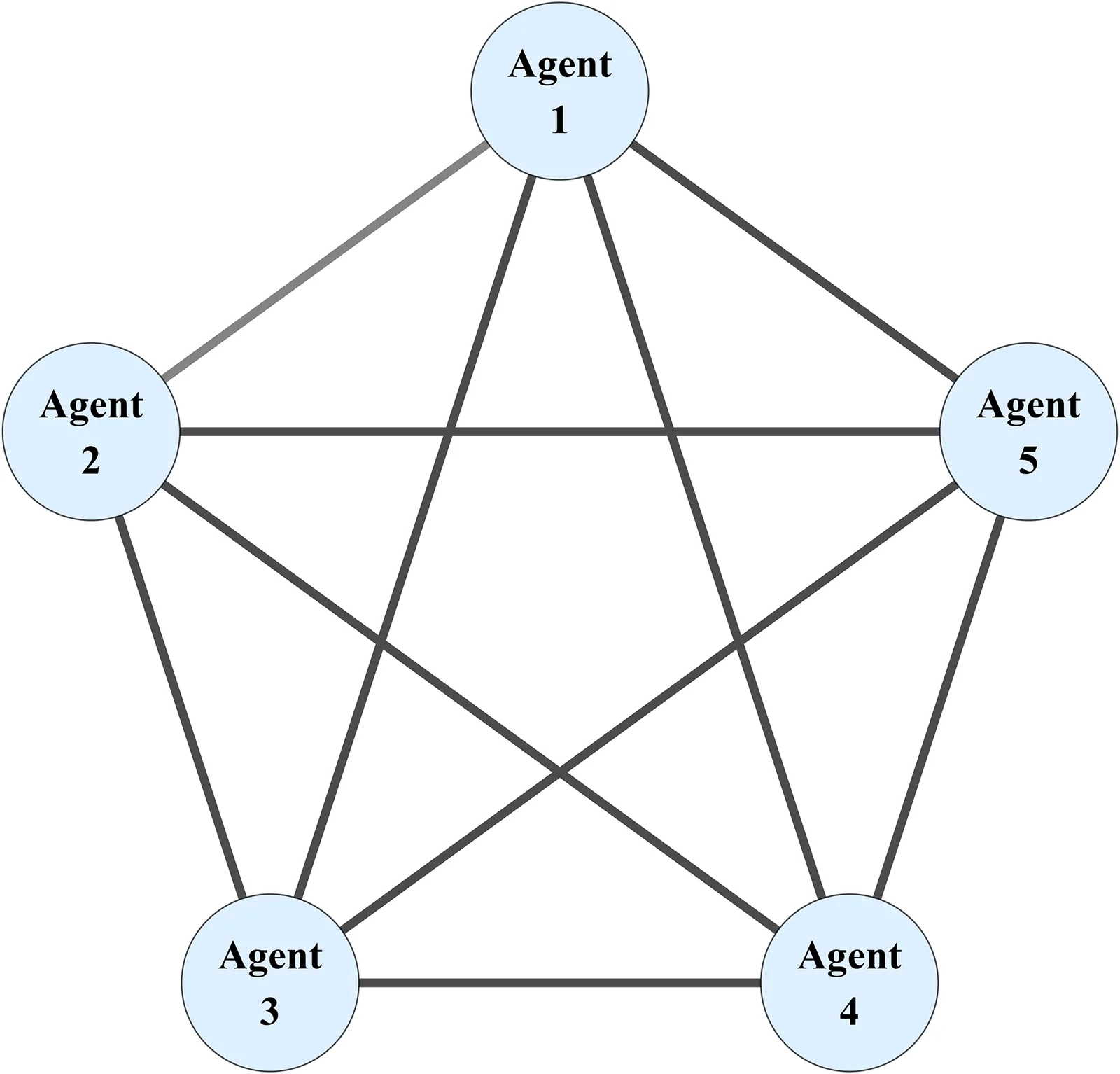
Representation of the swarm interaction topology modeled as a Complete Graph (*K*_5_). The graph consists of five nodes representing swarm individuals, with fully connected edges indicating that every agent communicates with every other agent to compute the global centroid.

Mathematical formalization of swarm coordination typically employs either Eulerian or Lagrangian approaches [13]. Focusing on individual agent dynamics, the Lagrangian (or microscopic) framework represents the state trajectories of distinct robots using ordinary differential equations [14]. By defining explicit attractive and repulsive forces between specific agents, the Lagrangian formulation allows for the direct tuning of local interactions required to achieve global objectives, such as obstacle circumnavigation and multi-agent task allocation [13]. Within this framework, coordination is generally regulated by the fundamental flocking rules proposed by Reynolds: separation to avoid collisions, alignment to synchronize movement, and cohesion to preserve group integrity [15]. The application of these rules depends significantly on the underlying communication architecture, which is often examined through algebraic graph theory [16]. The interaction topology determines the flow of information throughout the swarm. While some systems employ sparse networks, others assume a complete graph topology ($K_{n}$), where the graph Laplacian supports robust consensus algorithms. This connectivity enables the swarm to instantaneously compute global properties, such as the swarm centroid, to guarantee strong cohesion and synchronization during consensus-based rendezvous tasks [16]. A visual representation of this topology for a five-agent system is provided in Fig 1.

Several methodologies exist to achieve coordinated behaviors. Consensus algorithms use algebraic graph theory to ensure agents converge to shared states such as heading or velocity [16]. While optimization and learning techniques, including Particle Swarm Optimization (PSO) [17] and Reinforcement Learning (RL) [18], function effectively in high-dimensional spaces, their stochastic or data-driven nature limits their ability to provide the strict safety guarantees required for urban operations. The Artificial Potential Field (APF) method remains a common approach due to its low computational cost and energy-based structure [19, 20], although it also has limitations such as local minima. Standard heuristic APF approaches often lack a rigorous framework for proving asymptotic stability, leading to oscillations near obstacles. The Lyapunov-based Control Scheme (LbCS), a modified APF approach, addresses these stability issues. By defining a scalar energy function that accounts for target attraction and obstacle avoidance, the LbCS ensures the system follows a gradient that guarantees asymptotic stability via the direct method of Lyapunov, preventing oscillatory behavior [13,21,22].

The Segway Personal Transporter serves as the primary platform for this research, which is a two-wheeled, self-balancing vehicle characterized by inverted pendulum dynamics [23]. The Segway is highly suitable for urban mobility tasks [24,25]. Unlike larger autonomous vehicles, Segways can navigate narrow corridors and pedestrian-heavy zones. This extends the effective service coverage area within a city. These platforms are subject to non-holonomic constraints that restrict instantaneous motion strictly to the direction of the wheels [26,27]. This under-actuated characteristic complicates standard separation and cohesion rules, as lateral displacement requires prior reorientation. This under-actuated characteristic complicates standard separation and cohesion rules, as lateral displacement requires prior reorientation. Standard APF literature often simplifies the operating environment by treating obstacles solely as static disks. This modeling approach neglects complex geometric constraints, such as line-based boundaries, and fails to account for independent dynamic obstacles. Real-world urban environments feature complex geometries such as walls, curbs, and corridors. These features require line-based obstacle-avoidance strategies rather than simple point repulsion. A gap remains in the literature concerning rigorous, stability-guaranteed control schemes for non-holonomic swarms operating in cluttered environments. Many existing approaches rely on velocity-based controllers. When applied to dynamic platforms with significant inertia and torque limitations, these controllers can induce erratic motion [22,28].

To address these limitations, this paper proposes a rigorous control framework for a homogeneous swarm of Segway robots utilizing the LbCS. For under-actuated platforms like the Segway, embedding these potentials within a Lagrangian framework is essential, as it allows interaction objectives to be directly mapped to acceleration-level commands that respect the platform’s dynamic constraints. While recent work by Chand et al. (2025) established stabilizing acceleration-based controllers for a *single* Segway [28], prior swarm implementations have predominantly relied on instantaneous velocity commands [13,22], effectively treating agents as massless kinematic points. Bridging this gap, this work derives decentralized, continuous, non-linear acceleration-based controllers for the individuals of a swarm. By regulating the rate of change of velocity, these controllers inherently account for system inertia, ensuring smooth, time-invariant trajectories that are physically realizable for mechanically sensitive, self-balancing platforms. The proposed decentralized strategy operates under the assumption of a complete communication topology ($K_{n}$), where real-time information exchange regarding the swarm centroid facilitates robust cohesion and consensus-based rendezvous.

To resolve these limitations, a rigorous control framework is proposed for a homogeneous swarm of Segway robots utilizing the LbCS. Embedding these potentials within a Lagrangian framework is essential for under-actuated platforms. This approach maps interaction objectives directly to acceleration-level commands, thereby following the platform’s dynamic constraints. Recent research by Chand et al. (2025) established stabilizing acceleration-based controllers for a *single* Segway [28]. In contrast, prior swarm implementations predominantly rely on instantaneous velocity commands [13,22]. These velocity-based models effectively treat agents as massless kinematic points. To address this deficiency, decentralized, continuous, and nonlinear acceleration-based controllers are derived for the swarm’s individual agents. These controllers govern the rate of change of velocity to inherently account for system inertia. This governance guarantees smooth, time-invariant trajectories that are physically realizable for mechanically sensitive, self-balancing platforms. The decentralized strategy assumes a complete communication topology ($K_{n}$). Continuous exchange of information about the swarm centroid ensures robust cohesion and consensus-based rendezvous. The primary contributions of this paper are summarized as follows:

1. **Design of Distributed Acceleration Controllers:** A new set of stabilizing, continuous, non-linear acceleration controllers is designed for the agents of a Segway swarm. Although the physical implementation of the system utilizes a central processing unit to compute and transmit commands, the control laws themselves are mathematically *distributed*. Building upon the single-agent acceleration framework established in [28] and distinguishing this framework from prior velocity-based studies [13], this approach directly governs acceleration to account for system inertia, ensuring smooth, physically realizable motion for under-actuated platforms.
2. **Enhanced Collision Avoidance Scheme:** An advanced collision avoidance scheme is formulated to manage inter-agent interactions alongside complex environmental constraints. By incorporating both heterogeneous static boundaries (disks and lines) and independent dynamic obstacles, the swarm is equipped to navigate realistic, time-varying smart city geometries using a Minimum Distance Technique [29].
3. **Validation of Complex Emergent Behaviors:** The theoretical framework is validated through four progressively complex simulation scenarios. The results demonstrate the capacity of the system to execute advanced behaviors, including spontaneous formation plasticity to escape non-convex traps, decentralized rendezvous, and real-time reactive split-and-merge maneuvers to safely bypass non-yielding moving entities.

The remainder of this paper is structured as follows: Section 2 provides a comprehensive literature review. Section 3 presents the problem formulation, specific control objectives, and the kinematic modeling of the augmented system. Section 4 details the Lyapunov-based Control Scheme (LbCS) and the derivation of the distributed acceleration controllers. Section 5 outlines the numerical simulation results across four progressively complex scenarios. Section 6 provides a detailed discussion of the framework’s advancements and limitations. Finally, Section 7 concludes the paper. The formal mathematical proofs for system convergence and asymptotic controllability are detailed in the Appendix.

## 2 Literature review

The domain of autonomous multi-robot systems encompasses a wide range of methodologies, from distributed artificial intelligence to behavioral heuristics. To ensure theoretical coherence, a strictly defined scope is established, prioritizing Lagrangian formulations and the LbCS methodology. Alternative control paradigms are excluded to focus exclusively on agent-based mechanisms for acceleration-level derivation, which constitute the core theoretical foundation of this work. Within these boundaries, the existing literature is organized into three principal pillars: bio-inspired swarm coordination, mathematical modeling of collective dynamics, and navigational control strategies. This structured review establishes the theoretical basis for the proposed framework and identifies specific research gaps related to acceleration-level control in complex geometric environments.

### 2.1 Bio-inspired swarm control and Reynolds’ Rules

The foundational model for decentralized coordination is derived from Reynolds’ “boids” simulation, which demonstrated that complex aggregate motion emerges from three local heuristics: *separation*, *alignment*, and *cohesion* [15]. In robotic swarms, these rules are implemented by sensing neighbors within a finite radius and updating motion commands based on local positions and velocities [30]. Concretely, separation enforces a minimum inter-agent distance to prevent collisions; alignment synchronizes velocity vectors; and cohesion drives agents toward the local centroid to maintain group integrity.

While attractive for their simplicity, directly porting these abstraction-heavy rules to physical platforms such as Segways poses significant challenges. Real-world implementation must account for sensing noise, communication latency, and non-holonomic kinematic constraints, which prevent instantaneous lateral motion. To address these physical realities, researchers have explored adaptive weighting schemes, in which the influence of separation versus cohesion varies dynamically with environmental density [30], and evolutionary optimization methods to automatically tune flocking parameters [31]. These heuristic approaches frequently lack rigorous mathematical proofs of stability, leaving the system vulnerable to oscillatory behavior in cluttered spaces.

### 2.2 Lagrangian swarm models

To provide a rigorous mathematical basis for coordination, the literature distinguishes between Eulerian (continuum) and Lagrangian (agent-based) modeling approaches [14]. In the Eulerian approach, the swarm is considered a continuum described by its density in one-, two-, or three-dimensional space. In contrast, the Lagrangian approach is an individual-based method that explicitly studies the state (position, instantaneous velocity, and instantaneous acceleration) of each individual and its relationship with other individuals in the swarm. Within this framework, the velocity and acceleration of each agent can be directly influenced by its spatial coordinates. The planar Lagrangian swarm model developed in this research is based on the principle that swarming results from an interplay between long-range attraction and short-range repulsion among individuals. A common design paradigm combines these short-range repulsion potentials with long-range attraction potentials [30]. Repulsive terms, often modeled as inverse barrier functions, increase sharply at small separations to guarantee collision avoidance, while attractive terms guide agents toward the swarm centroid or target. Tuning the relative gains of these potentials governs the swarm’s density and formation shape.

### 2.3 Navigation and obstacle avoidance strategies

#### 2.3.1 Artificial Potential Fields (APF).

For reactive navigation in cluttered environments, the APF method remains a standard due to its low computational cost [19,20]. By assigning repulsive forces to obstacles and attractive forces to targets, APF provides a unified mechanism for path planning. Classical APF formulations frequently generate local minima, where the net force vanishes at non-target configurations, potentially trapping the agent [32].

Recent literature has proposed numerous improvements to mitigate these local-minima traps, including adaptive potential fields that dynamically adjust repulsive forces [33] and optimization-based tuning via metaheuristics such as PSO to escape local traps [17]. Hybridization with control strategies like Sliding Mode Control (SMC) has also been explored to enhance tracking precision [34]. Recent studies also introduce probabilistic and collaborative mechanisms to overcome classical APF limitations. In the context of multi-agent coordination, Falomir et al. (2018) introduced an improved APF mobility model for UAV swarms wherein agents actively share detected obstacle data, enhancing global path planning beyond individual sensor horizons [35]. Similarly, Almaameri and Blázovics (2025) developed a Quantum-Enhanced Artificial Potential Field (QEAPF) for drones, demonstrating that adaptive parameter tuning coupled with quantum-inspired exploration significantly accelerates formation convergence and improves disturbance rejection [36]. While recent advances have incorporated adaptive, optimization-based, and learning-based enhancements to Artificial Potential Field (APF) methods, many of these approaches emphasize navigation performance without providing rigorous theoretical guarantees of asymptotic stability and convergence. Such guarantees are particularly important for safety-critical autonomous transportation systems operating in complex urban environments.

#### 2.3.2 The Lyapunov-based control scheme (LbCS).

A rigorous evolution of the APF method is the LbCS. In contrast to heuristic APF approaches that aggregate attractive and repulsive forces, LbCS systematically derives control laws directly from the gradient of a Lyapunov function, the total potential. This formulation ensures that the time derivative of the system’s energy function remains negative semi-definite [13]. The framework provides a formal mathematical guarantee of asymptotic stability and convergence to the equilibrium state using the direct Lyapunov method, addressing the stability limitations inherent in classical potential field methods.

Foundational work by Kumar et al. (2021) demonstrated the efficacy of LbCS for swarm organization, using velocity-based controllers to generate complex behaviors, such as elliptical formations and tunneling, for Unmanned Aerial Vehicles (UAVs) [13]. Addressing the specific dynamics of personal transporters, Kumar et al. (2023) subsequently extended the framework to a single Segway agent; this implementation, relying on instantaneous velocity commands, can induce jerky motion [22]. In parallel, Sharma et al. (2023) advanced the methodology by deriving acceleration-based controllers for a compartmentalized robot navigating complex line-based constraints [37]. Most recently, Chand et al. (2025) successfully established stabilizing acceleration-based controllers for a single Segway, ensuring smooth motion and asymptotic stability [28].

Despite this considerable progress in LbCS methodologies, several important research gaps remain. Existing LbCS-based swarm studies have primarily employed velocity-based controllers, which are appropriate for idealized kinematic agents such as UAVs or point-mass robots but do not adequately capture the dynamic characteristics of under-actuated Segway platforms. Since Segway robots exhibit inertia and nonholonomic constraints, velocity-based control may result in abrupt motion changes that are physically unattainable. Although acceleration-based LbCS has recently been developed for a single Segway robot, its extension to cooperative multi-agent systems has not yet been investigated.

Another limitation of the current literature is its representation of the operating environment. Most existing studies assume simplified obstacle geometries consisting primarily of disk obstacles, which do not accurately reflect the complexity of real urban environments. Practical smart-city applications require autonomous robots to navigate around heterogeneous static structures, including walls, corridors, pavements, and other elongated boundaries, as well as conventional disk-shaped obstacles. Extending acceleration-based control from a single robot to a swarm introduces additional challenges related to swarm cohesion, inter-agent collision avoidance, decentralized coordination, and consensus-based navigation, all while maintaining system stability in the presence of static and dynamic obstacles.

A mathematically rigorous framework combining physically realistic acceleration-level control with formal Lyapunov stability analysis is required for cooperative Segway swarms. To address these limitations, a distributed acceleration-based control framework is developed for a homogeneous swarm of Segway robots operating in heterogeneous smart-city environments. The proposed methodology extends the LbCS from single-agent systems to cooperative multi-agent navigation by integrating swarm cohesion, inter-agent collision avoidance, static disk and line obstacle avoidance, and dynamic obstacle evasion within a unified mathematical framework. Unlike previous studies, the proposed controllers are derived at the acceleration level, thereby accounting for the physical dynamics of Segway platforms while providing rigorous mathematical proofs of stability, convergence, and asymptotic controllability.

These contributions bridge the gap between existing theoretical developments and the practical deployment of autonomous Segway swarms in realistic urban environments.

### 2.4 Research gap and contributions

Table 1 presents a comparative analysis of key studies in the field, highlighting the specific gaps addressed by this research. While prior works have addressed UAV swarms or single Segways, there remains a lack of rigorous, acceleration-based control frameworks for Segway swarms operating in environments with heterogeneous (disk and line) shaped static constraints.

**Table 1:** Comparative Analysis of Key Studies and Advancements.

Paper	Contribution	Advancement by This Work
**Khatib (1986)** [19]	Introduced Artificial Potential Fields (APF) for real-time obstacle avoidance.	Replaces heuristic force summation with a rigorous Lyapunov framework ensuring asymptotic stability.
**Olfati-Saber (2006)** [38]	Established graph-theoretic framework for flocking and consensus.	Extends abstract point-mass consensus to physical, under-actuated Segway platforms with non-holonomic constraints.
**Kumar et al. (2021)** [13]	Developed velocity-based LbCS for UAV swarms (disk obstacles).	Derives acceleration-based controllers; extends environmental modeling to complex line obstacles (walls).
**Kumar et al. (2023)** [22]	Established velocity-based LbCS for a single Segway.	Upgrades to acceleration-level control; scales to a multi-agent swarm with collision avoidance.
**Sharma et al. (2023)** [37]	Applied acceleration LbCS to a rigid multi-unit robot.	Distributed controllers for the independent agents of a swarm, enabling flexible formation changes.
**Chand et al. (2025)** [28]	Derived acceleration controllers for single Segway navigation.	Scales the acceleration framework to a multi-agent swarm (*n* agents) with centroid cohesion and active dynamic obstacle avoidance.

The comparison highlights the distinct contributions of the present study. By moving from velocity to acceleration control and from simple disk worlds to complex line-based geometries, this work bridges the gap between theoretical swarm stability and practical urban mobility requirements.

## 3 Problem formulation and system modeling

The operational efficiency of future urban environments relies on the deployment of intelligent and autonomous mobility systems. While single personal transporters like the Segway present a solution for last-mile transit, their utility is maximized through the coordinated operation of autonomous fleets. This work transitions from the control of an individual agent to the more complex challenge of collective navigation, addressing the safety and coordination issues inherent in multi-robot systems.

Within a high-density Smart City, a fleet of personal transporters must navigate a shared, cluttered environment. Unlike open fields typical of UAV operations, this workspace is strictly constrained by physical infrastructure, such as pavements, building walls, and corridors, as well as distinct static obstacles, such as pillars or street furniture. The agents in this system are Segway Personal Transporters, characterized by underactuated, nonholonomic kinematic constraints.

### 3.1 The scenario: Decentralized Urban swarms

Consider a high-density urban environment where a fleet of personal transporters must navigate a shared, constrained workspace. Unlike macroscopic traffic flow models, this system is analyzed through a Lagrangian framework, treating the swarm as a collection of discrete, microscopic agents. The agents in this system are Segway Personal Transporters, characterized by underactuated, nonholonomic kinematic constraints.

The specific problem addressed is the safe and coordinated navigation of a homogeneous swarm of n∈ℕ Segway robots operating within a constrained two-dimensional workspace $𝒲\subset {\mathbb{R}}^{2}$. The environment is populated by heterogeneous static constraints, geometrically modeled as disks (representing pillars, wells, or roundabouts) and line segments (representing walls, curbs, or corridors), as well as independent dynamic obstacles representing moving pedestrians or uncooperative vehicles. The fundamental challenge is to derive a distributed control law that follows the interplay between long-range attractive forces and short-range repulsive forces. This control law must drive the swarm from an arbitrary set of initial states to a desired equilibrium configuration, a process formally described as consensus-based rendezvous, while strictly satisfying kinematic feasibility and safety guarantees in a time-varying environment.

### 3.2 System modeling assumptions

To ensure the feasibility of the control design while maintaining practical relevance, the following assumptions are established based on standard swarm theoretic norms:

1. **Lagrangian Formulation:** The swarm is treated as a set of distinct agents indexed by i∈{1,...,n}, where the state of each agent is governed by its own ordinary differential equations.
2. **Complete Interaction Topology:** The communication network is modeled as a complete graph ($K_{n}$). Every agent *i* has access to the state information of every other agent *j*, allowing for the instantaneous computation of the swarm centroid to enforce cohesion.
3. **Semi-Dynamic Environment:** The obstacle field comprises both time-invariant geometric constraints (static disks and lines) and time-varying, independent dynamic obstacles.
4. **Flat Terrain:** The motion is restricted to the horizontal plane, neglecting vertical displacement or pitch/roll dynamics beyond the scope of planar navigation.

To formalize the problem, let *z*_1_ and *z*_2_ denote the orthogonal spatial coordinates representing the horizontal and vertical axes of the 2D Cartesian workspace 𝒲, respectively. The following definitions are established:

**Definition 3.1 (Swarm Target).** *The target for the Segway swarm’s centroid is a disk with center* $\left(\tau_{1},\tau_{2}\right)$ *and radius* $r_{wc}$*. It is described as the set:*


$$\begin{array}{c} 𝒯:=\{(z_{1},z_{2})\in {\mathbb{R}}^{2}:(z_{1}-\tau_{1}{)}^{2}+(z_{2}-\tau_{2}{)}^{2}\leq r_{wc}^{2}\}. \end{array}$$ (1)

**Definition 3.2 (Agent State).** *The state of the i*th *Segway in the swarm, for* i∈{1,...,n}*, is described by its Cartesian position* $\left(x_{i},y_{i}\right)$*, its orientation angle* $\theta_{i}$*, and the angular velocities of its wheels,* $v_{R_{i}}$ *and* $v_{L_{i}}$*.*

**Definition 3.3 (Static Disk Obstacle).** *The k*th *solid stationary obstacle is a disk with center* $\left(o_{x_{k}},o_{y_{k}}\right)$ *and radius* $r_{k}>0$*. It is described as the set:*


$$\begin{array}{c} {𝒪}_{D_{k}}:=\{(z_{1},z_{2})\in {\mathbb{R}}^{2}:(z_{1}-o_{x_{k}}{)}^{2}+(z_{2}-o_{y_{k}}{)}^{2}\leq r_{k}^{2}\}. \end{array}$$ (2)

**Definition 3.4 (Static Line Obstacle).** *The m*th *line obstacle is the line segment from the point* $\left(e_{m_{1}},f_{m_{1}}\right)$ *to the point* $\left(e_{m_{2}},f_{m_{2}}\right)$ *and is described as the set:*


$$\begin{array}{c} {𝒪}_{L_{m}}:=\left\{(z_{1},z_{2})\in {\mathbb{R}}^{2}:(z_{1}-e_{m_{1}}-\lambda (e_{m_{2}}-e_{m_{1}}){)}^{2}+(z_{2}-f_{m_{1}}-\lambda (f_{m_{2}}-f_{m_{1}}){)}^{2}=0\right\} \end{array}$$ (3)

where λ∈[0,1].

**Definition 3.5** (Swarm Centroid). *The centroid of the swarm of* n∈ℕ *Segway robots is denoted by* ${𝐱}_{c}=(\bar{x},\bar{y})$*, where*


$$\begin{array}{c} \bar{x}=\frac{1}{n}\sum_{j=1}^{n}x_{j},\;\text{and}\;\bar{y}=\frac{1}{n}\sum_{j=1}^{n}y_{j}. \end{array}$$ (4)

**Definition 3.6** (Dynamic Obstacle). *The h*th *dynamic obstacle, for* h∈{1,...,p}*, is modeled as a moving rigid body residing in a disk with a time-varying center* $\left(x_{d_{h}},y_{d_{h}}\right)$ *and radius* $r_{d_{h}}>0$*. It is described as the set:*


$$\begin{array}{c} {𝒪}_{DO_{h}}:=\{(z_{1},z_{2})\in {\mathbb{R}}^{2}:(z_{1}-x_{d_{h}}{)}^{2}+(z_{2}-y_{d_{h}}{)}^{2}\leq r_{d_{h}}^{2}\}. \end{array}$$ (5)

**Definition 3.7** (Dynamic Obstacle Target). *The target for the h*th *dynamic obstacle is a stationary disk with center* $\left(\tau_{h_{1}},\tau_{h_{2}}\right)$ *and radius* $r_{\tau_{h}}>0$*. It is described as the set:*


$$\begin{array}{c} {𝒯}_{DO_{h}}:=\{(z_{1},z_{2})\in {\mathbb{R}}^{2}:(z_{1}-\tau_{h_{1}}{)}^{2}+(z_{2}-\tau_{h_{2}}{)}^{2}\leq r_{\tau_{h}}^{2}\}. \end{array}$$ (6)

### 3.3 Kinematic model of segway robot

**Fig 2:**
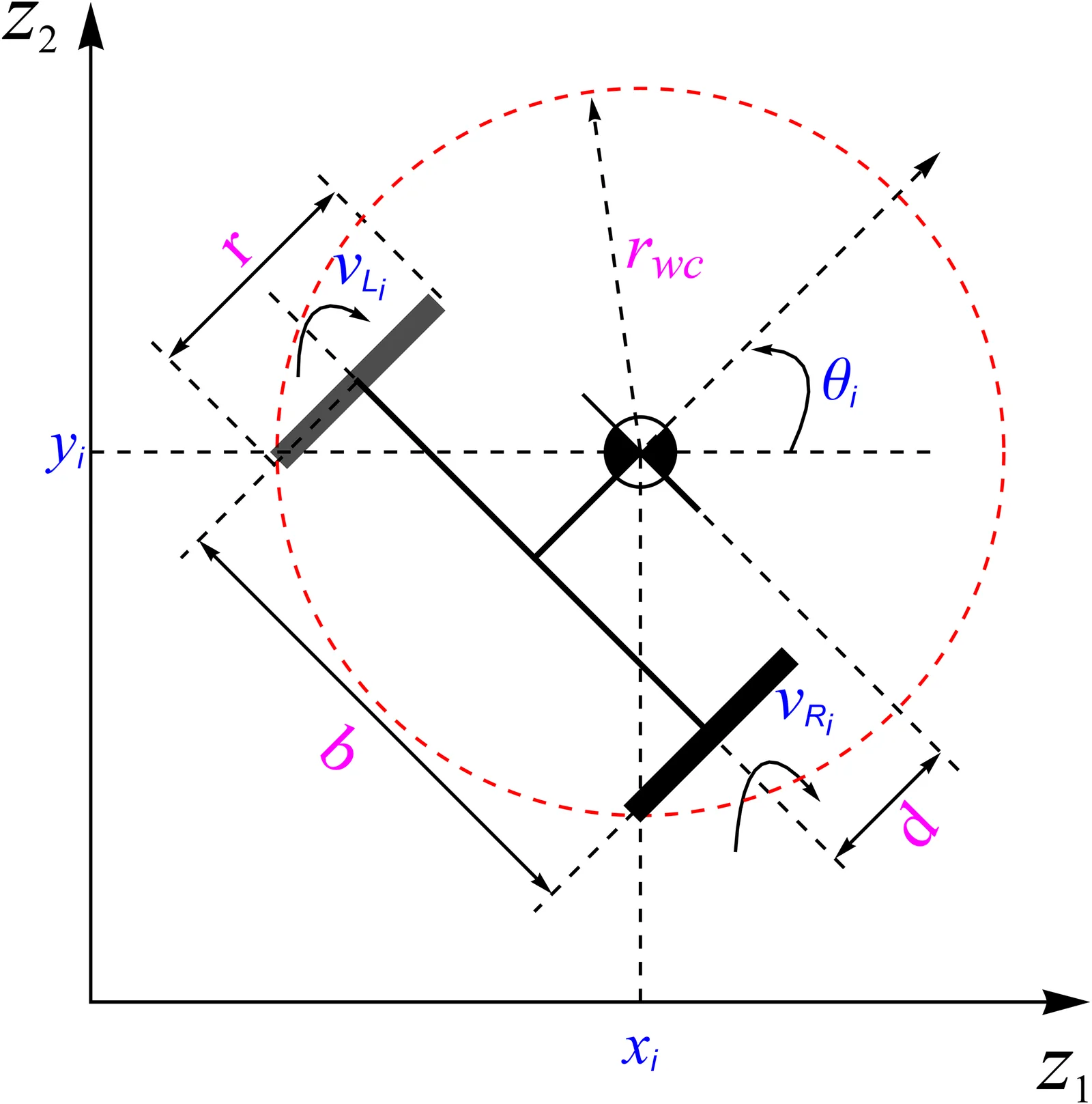
Schematic diagram of the *i*th Segway robot modified from [22].

The system under consideration is a swarm composed of n∈ℕ autonomous Segway robots. The kinematic model governing the *i*th agent, as illustrated in Fig 2, is a modification of the formulation established in [22].

The position of the *i*th Segway robot is defined by the coordinates $\left(x_{i},y_{i}\right)$, which represent the robot’s center of mass (located at the handlebar). The orientation is given by the heading angle $\theta_{i}$ relative to the horizontal axis. Geometrically, the agent is characterized by a wheel track width of *b* and a wheel radius of *r*/2. The angular velocities of the left and right wheels for the *i*th agent are denoted by $v_{L_{i}}$ and $v_{R_{i}}$, respectively. To ensure operational safety, each agent is enclosed within a circular protective region of radius $r_{wc}$ for collision avoidance.

The motion of the Segway agents is governed by non-holonomic constraints arising from the assumption of pure rolling without slip. Physically, this imposes two specific restrictions on the wheel-ground interaction:

1. **No Lateral Slip:** The instantaneous velocity of the wheel contact point in the lateral direction must be zero, preventing the robot from sliding sideways.
2. **Pure Rolling:** The longitudinal velocity of the wheel axis is determined strictly by the angular rotation of the wheels.

These constraints couple the robot’s translational velocity components $\left({\dot{x}}_{i},{\dot{y}}_{i}\right)$ to its orientation $\theta_{i}$ and wheel velocities. Since the system state tracks the center of mass (offset by distance *d* from the wheel axis), the no-slip condition enforces a kinematic coupling where the robot’s lateral motion is inextricably linked to its angular velocity. Solving these constraint equations yields the specific kinematic model described by the system of ordinary differential equations:


$$\begin{array}{c} \begin{array}{c} {\dot{x}}_{i}=c(v_{L_{i}}(b\cos\theta_{i}+d\sin\theta_{i})+v_{R_{i}}(b\cos\theta_{i}-d\sin\theta_{i}))\\ {\dot{y}}_{i}=c(v_{L_{i}}(b\sin\theta_{i}-d\cos\theta_{i})+v_{R_{i}}(b\sin\theta_{i}+d\cos\theta_{i}))\\ {\dot{\theta }}_{i}=c(v_{R_{i}}-v_{L_{i}})\\ {\dot{v}}_{R_{i}}=a_{R_{i}},\;{\dot{v}}_{L_{i}}=a_{L_{i}} \end{array}\} \end{array}$$ (7)

where:

- $a_{L_{i}}$, $a_{R_{i}}$: Angular accelerations of the left and right wheels of the *i*th Segway, respectively.
- *c* = *r* / (2*b*), where *r* is the wheel radius and *b* is the distance between the wheels.
- *d* is the distance from the handle to the centre of the axle connecting the wheels.

The control inputs for each Segway are the wheel accelerations, $a_{R_{i}}$ and $a_{L_{i}}$.

To model the moving entities within the workspace, each dynamic obstacle is treated as an independent point-mass system controlled by its acceleration. The motion of the *h*th dynamic obstacle, where h∈{1,...,p}, is described by its Cartesian position $\left(x_{d_{h}},y_{d_{h}}\right)$ and its translational velocity components $\left(v_{d_{h}},w_{d_{h}}\right)$.

The movement of each dynamic obstacle is governed by its own set of control inputs, specifically the accelerations $u_{1_{h}}$ and $u_{2_{h}}$ in the *x* and *y* directions, respectively. The kinematic model is defined by the following system of ordinary differential equations:


$$\begin{array}{c} \begin{array}{c} {\dot{x}}_{d_{h}}=v_{d_{h}},\;{\dot{y}}_{d_{h}}=w_{d_{h}},\\ {\dot{v}}_{d_{h}}=u_{1_{h}},\;{\dot{w}}_{d_{h}}=u_{2_{h}}. \end{array}\} \end{array}$$ (8)

#### 3.3.1 Global kinematic model.

The complete workspace consists of the autonomous swarm and the independent dynamic obstacles. By aggregating the non-holonomic kinematics of the *n* Segway agents and the translational kinematics of the *p* dynamic obstacles, the global kinematic model for the entire augmented system is defined by the following system of ordinary differential equations:


$$\begin{array}{c} \begin{array}{c} {\dot{x}}_{i}=c(v_{L_{i}}(b\cos\theta_{i}+d\sin\theta_{i})+v_{R_{i}}(b\cos\theta_{i}-d\sin\theta_{i}))\\ {\dot{y}}_{i}=c(v_{L_{i}}(b\sin\theta_{i}-d\cos\theta_{i})+v_{R_{i}}(b\sin\theta_{i}+d\cos\theta_{i}))\\ {\dot{\theta }}_{i}=c(v_{R_{i}}-v_{L_{i}})\\ {\dot{v}}_{R_{i}}=a_{R_{i}},\;{\dot{v}}_{L_{i}}=a_{L_{i}}\\ {\dot{x}}_{d_{h}}=v_{d_{h}},\;{\dot{y}}_{d_{h}}=w_{d_{h}}\\ {\dot{v}}_{d_{h}}=u_{1_{h}},\;{\dot{w}}_{d_{h}}=u_{2_{h}} \end{array}\} \end{array}$$ (9)

for all swarm agents i∈{1,...,n} and all dynamic obstacles h∈{1,...,p}.

#### 3.3.2 System state vectors.

To represent the complete environment, comprising the autonomous swarm and the independent dynamic obstacles, an augmented global state vector must be defined.

The state vector for the *i*th Segway swarm agent remains:


$$\begin{array}{c} {𝐱}_{s_{i}}=[x_{i},y_{i},\theta_{i},v_{R_{i}},v_{L_{i}}{]}^{T}\in {\mathbb{R}}^{5} \end{array}$$ (10)

The state vector for the *h*th dynamic obstacle is defined as:


$$\begin{array}{c} {𝐱}_{d_{h}}=[x_{d_{h}},y_{d_{h}},v_{d_{h}},w_{d_{h}}{]}^{T}\in {\mathbb{R}}^{4} \end{array}$$ (11)

The global state of the entire system, denoted by the augmented vector 𝐱, concatenates the states of all n∈ℕ Segway agents and all p∈ℕ dynamic obstacles:


$$\begin{array}{c} 𝐱={\left[\begin{array}{c} {𝐱}_{s_{1}}^{T},...,{𝐱}_{s_{n}}^{T},{𝐱}_{d_{1}}^{T},...,{𝐱}_{d_{p}}^{T} \end{array}\right]}^{T}\in {\mathbb{R}}^{5n+4p}. \end{array}$$ (12)

#### 3.3.3 Initial and equilibrium configurations.

The system initializes at time $t_{0}\geq 0$ from a specified condition ${𝐱}_{0}$. The initial state vector for the *i*th Segway agent is defined as:


$$\begin{array}{c} {𝐱}_{s_{i_{0}}}=[x_{i_{0}},y_{i_{0}},\theta_{i_{0}},v_{R_{i_{0}}},v_{L_{i_{0}}}{]}^{T}. \end{array}$$ (13)

The initial state for the entire swarm, ${𝐱}_{s_{0}}$, consists of the stacked individual initial vectors:


$$\begin{array}{c} {𝐱}_{s_{0}}=[{𝐱}_{s_{1_{0}}}^{T},...,{𝐱}_{s_{n_{0}}}^{T}{]}^{T}. \end{array}$$ (14)

Similarly, the initial state for the *h*th dynamic obstacle is:


$$\begin{array}{c} {𝐱}_{d_{h_{0}}}=[x_{d_{h_{0}}},y_{d_{h_{0}}},v_{d_{h_{0}}},w_{d_{h_{0}}}{]}^{T}, \end{array}$$ (15)

and the initial state for all *p* dynamic obstacles is the stacked vector:


$$\begin{array}{c} {𝐱}_{d_{0}}=[{𝐱}_{d_{1_{0}}}^{T},...,{𝐱}_{d_{p_{0}}}^{T}{]}^{T}. \end{array}$$ (16)

The global initial state for the complete augmented system is the concatenation of the swarm and dynamic obstacle initial states:


$$\begin{array}{c} {𝐱}_{0}=𝐱(t_{0})=[\begin{array}{c} {𝐱}_{s_{0}}\\ {𝐱}_{d_{0}} \end{array}]. \end{array}$$ (17)

The desired final configuration is the stable equilibrium point, ${𝐱}_{e}$, where all motion in the environment ceases and control objectives are satisfied. For the *i*th Segway agent, this state is given by:


$$\begin{array}{c} {𝐱}_{s_{i_{e}}}=[x_{i_{e}},y_{i_{e}},\theta_{i_{e}}^{*},0,0{]}^{T} \end{array}$$ (18)

where $\left(x_{i_{e}},y_{i_{e}}\right)$ and $\theta_{i_{e}}^{*}$ represent the final position and orientation satisfying the formation requirements. The equilibrium state for the entire swarm is:


$$\begin{array}{c} {𝐱}_{s_{e}}=[{𝐱}_{s_{1_{e}}}^{T},...,{𝐱}_{s_{n_{e}}}^{T}{]}^{T}. \end{array}$$ (19)

This state ${𝐱}_{s_{e}}$ incorporates a stable equilibrium where the swarm centroid aligns with the global target, implying:


$$\begin{array}{c} \sum_{i=1}^{n}(x_{i_{e}},y_{i_{e}})=(\tau_{1},\tau_{2}). \end{array}$$ (20)

For the *h*th dynamic obstacle, the equilibrium state means it has reached its specific target $\left(\tau_{h_{1}},\tau_{h_{2}}\right)$ with zero velocity:


$$\begin{array}{c} {𝐱}_{d_{h_{e}}}=[\tau_{h_{1}},\tau_{h_{2}},0,0{]}^{T}. \end{array}$$ (21)

The collective equilibrium for all dynamic obstacles is:


$$\begin{array}{c} {𝐱}_{d_{e}}=[{𝐱}_{d_{1_{e}}}^{T},...,{𝐱}_{d_{p_{e}}}^{T}{]}^{T}. \end{array}$$ (22)

Finally, the global equilibrium point for the entire augmented system is the stacked vector:


$$\begin{array}{c} {𝐱}_{e}=[\begin{array}{c} {𝐱}_{s_{e}}\\ {𝐱}_{d_{e}} \end{array}]. \end{array}$$ (23)

At this state ${𝐱}_{e}$, the swarm satisfies its formation and target constraints, all dynamic obstacles rest at their respective targets, and all velocities in the system are strictly zero.

#### 3.3.4 System representation.

The instantaneous accelerations governing both the Segway agents and the dynamic obstacles are determined by a combined feedback control law. This control law, denoted as 𝐮(𝐱), aggregates the required inputs for every agent within the workspace.

For the *i*th Segway agent, the control inputs are the angular accelerations of the right and left wheels, $a_{R_{i}}$ and $a_{L_{i}}$. For the *h*th dynamic obstacle, the control inputs are the translational accelerations $u_{1_{h}}$ and $u_{2_{h}}$. The unified control vector is defined as:


$$\begin{array}{c} 𝐮(𝐱)=[a_{R_{1}},a_{L_{1}},...,a_{R_{n}},a_{L_{n}},u_{1_{1}},u_{2_{1}},...,u_{1_{p}},u_{2_{p}}{]}^{T}\in {\mathbb{R}}^{2n+2p}. \end{array}$$ (24)

The dynamics of the complete environment consisting of the Segway swarm kinematics and the dynamic obstacle motions represented by equation (9) are represented by an augmented vector field E(𝐱). By defining the system’s vector field such that 𝐮(𝐱) is embedded within E(𝐱), the complete autonomous motion of the system can be expressed in the compact state-space form:


$$\begin{array}{c} \dot{𝐱}=E(𝐱),\;𝐱(t_{0})={𝐱}_{0}. \end{array}$$ (25)

### 3.4 Control objectives

To solve the described problem, the following specific control objectives are established:

1. To formulate a novel set of nonlinear, time-invariant, continuous acceleration-based controllers that govern the motion of each agent within the autonomous Segway swarm.
2. To achieve reliable, coordinated navigation of the swarm from a given set of initial configurations to a desired final swarm centroid configuration in an environment containing both static geometric constraints and moving entities.
3. To employ the LbCS framework to formally guarantee inter-agent collision avoidance, static obstacle avoidance, and dynamic obstacle evasion, while simultaneously enforcing swarm cohesion.
4. To validate the system’s performance through rigorous mathematical proofs of stability, asymptotic controllability, and convergence for the entire multi-agent system.

## 4 Design of Lyapunov-based acceleration controllers

The control architecture presented in this paper is founded on the LbCS, a model-based framework that provides a systematic method for designing stabilizing controllers for complex nonlinear systems [13,22]. As a rigorous implementation of the APF methodology, LbCS is used to generate smooth, collision-free stabilizing nonlinear trajectories for autonomous systems [13,22,28,37].

The core of the scheme involves constructing a scalar total potential, energy-like function, the Lyapunov function, over the system’s entire state space. This function creates a virtual potential field where the desired final state of the system corresponds to a unique global minimum. For the multi-agent system considered here, this potential field is composed of several distinct components:

- An **attractive potential** (rendezvous), which draws the swarm’s collective centroid towards the global target (alignment).
- **Repulsive potentials**, which repel each agent away from the boundaries of static obstacles and the trajectories of time-varying dynamic entities in the environment.
- **Inter-agent repulsive potentials** (separation), acting as short-range repulsion forces between every pair of agents to prevent internal collisions.
- **Cohesion potentials** (long-range attraction), which ensure individual agents remain aggregated around the swarm’s centroid.

These individual potentials are mathematically summed to construct a composite Lyapunov function, L(𝐱), for the entire swarm. The system’s controllers are derived directly from the negative gradient of this function, −∇L(𝐱). This gradient vector points in the direction of steepest descent on the potential energy surface, continuously driving the system toward the target equilibrium. The gradient dictates the required acceleration inputs for each Segway agent.

A primary strength of the LbCS framework lies in its ability to explicitly incorporate mechanical system limitations. A known limitation of potential field methods is the system’s susceptibility to local minima, where the potential gradient is zero at a non-target location [13,22].

**Fig 3:**
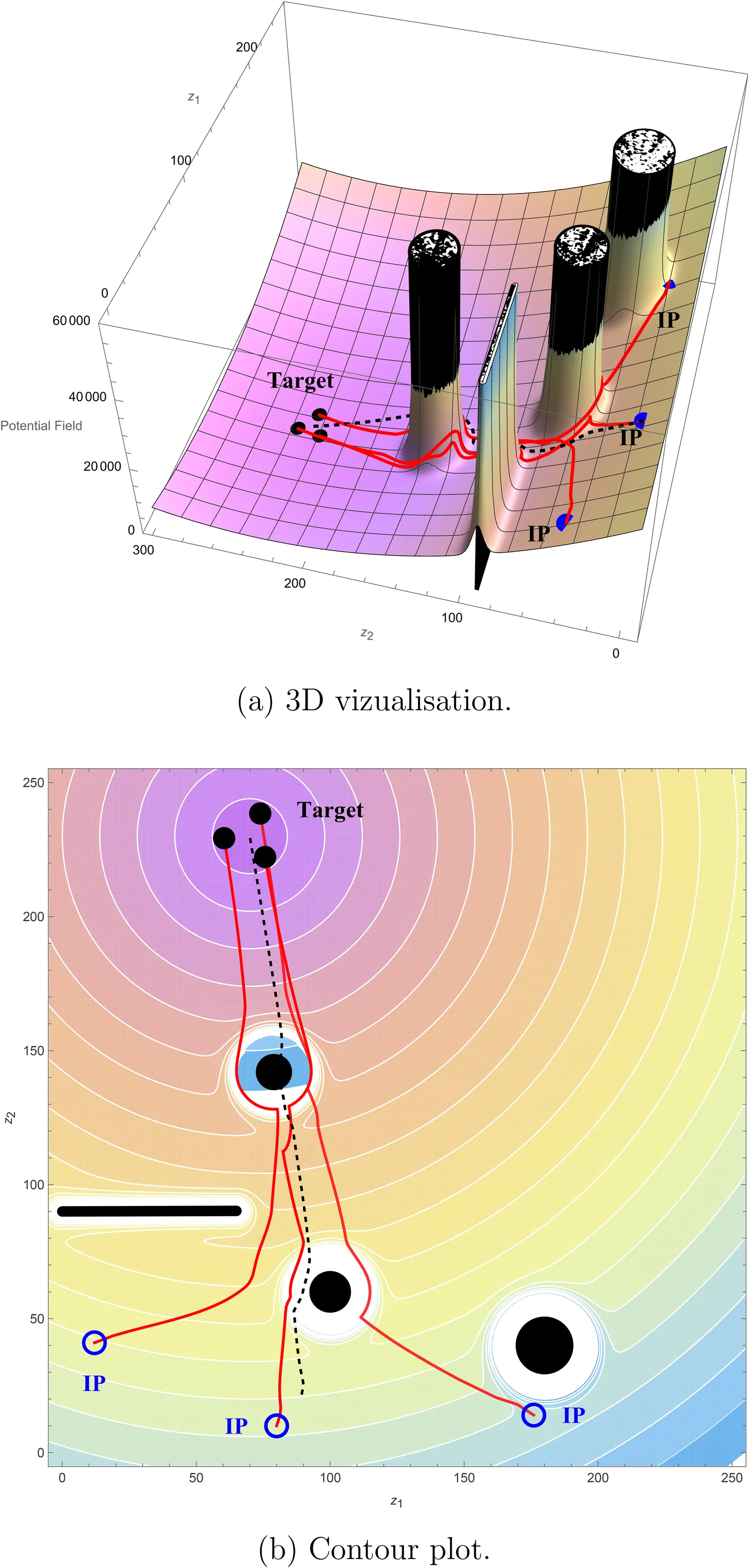
Visualization of the LbCS framework applied to swarm navigation. (a) The 3D potential energy landscape, where the target acts as the global minimum (attractive basin). The geometric obstacles (three disks and one line segment) are mathematically represented as high-potential peaks approaching infinity, creating strict repulsive barriers. (b) The corresponding 2D contour projection. Agents begin at distributed Initial Positions (IP, denoted by blue circles) and perform continuous gradient descent. The solid red lines depict the distinct, collision-free trajectories of the individual agents as they actively evade the constraints (visualized by the repulsive blue/white halos). The dashed black line explicitly traces the swarm’s centroid path, confirming successful global convergence and inter-agent spatial separation at the Target (black markers).

Fig 3 provides a numerical validation of this framework using a swarm of three Segway agents navigating a complex environment containing three static disk obstacles and one linear barrier. As established in foundational LbCS literature [13,22], the 3D visualization in Fig 3(a) depicts the total potential energy surface. On this surface, the repulsive barrier functions associated with the static constraints manifest as high-potential peaks that approach infinity at the obstacle boundaries, while the target attraction function creates the global minimum valley. This geometric representation highlights the cohesive nature of the control scheme: the inter-agent and obstacle repulsive potentials force the agents to locally deviate and climb slightly up the potential slopes to avoid collisions, while the cohesion potential continuously acts as an elastic restoring force, pulling them back toward the centroid’s path.

Furthermore, the 2D contour plot in Fig 3(b) shows a top-down perspective of the swarm’s spatial evolution along the equipotential lines. The agents originate from distinct Initial Positions (IP) and follow the orthogonal gradient descent towards their global minimum. The contour spacing visually demonstrates the varying intensity of the repulsive fields, successfully forcing the swarm to split and navigate around the black linear and disk obstacles before re-forming to rendezvous safely at the target.

### 4.1 Design of acceleration controllers

The swarm of n∈ℕ Seqway’s are cluttered with q∈ℕ line and p∈ℕ dynamic obstacles. The swarm should successfully navigate the workspace to reach the equilibrium. The primary objective of this section is to design the feedback control law 𝐮(𝐱), embedded within the vector field E(𝐱). The controllers are synthesized to guarantee that the swarm system dynamics $\dot{𝐱}=E(𝐱)$ remain stable and that the state 𝐱(t) converges to the desired equilibrium configuration ${𝐱}_{e}$. To achieve this, the acceleration controllers for each agent are systematically derived using a composite Lyapunov function, constructed from a set of attractive and repulsive potential functions. The following functions are the foundational components for constructing the total potential.

#### 4.1.1 Target attraction and auxiliary functions.

To guide the swarm’s centroid to its target $\left(\tau_{1},\tau_{2}\right)$, the following target attraction function is utilized:


$$\begin{array}{c} T(𝐱)=\frac{1}{2}\left((\bar{x}-\tau_{1}{)}^{2}+(\bar{y}-\tau_{2}{)}^{2}+\sum_{i=1}^{n}(v_{L_{i}}^{2}+v_{R_{i}}^{2})\right). \end{array}$$ (26)

#### 4.1.2 Swarm cohesion function.

To maintain long-range attraction and the cohesion of the swarm, the function $G_{i}(𝐱)$ attracts each Segway towards the swarm’s centroid:


$$\begin{array}{c} G_{i}(𝐱)=\frac{1}{2}\left((x_{i}-\bar{x}{)}^{2}+\delta (y_{i}-\bar{y}{)}^{2}\right). \end{array}$$ (27)

The control parameter δ>0 determines the eccentricity of the swarm’s formation by weighting the attraction in the *y*-direction relative to the *x*-direction. Adjusting δ allows the swarm to assume different spatial configurations, such as circular, elliptical, or linear formations.

#### 4.1.3 Inter-Agent Collision Avoidance.

To ensure short-range repulsion, the function


$$\begin{array}{c} A_{ij}(𝐱)=\frac{1}{2}\left((x_{i}-x_{j}{)}^{2}+(y_{i}-y_{j}{)}^{2}-(2r_{wc}{)}^{2}\right),\;\forall i\neq j \end{array}$$ (28)

is defined to prevent collision between the *i*th and *j*th agents.

#### 4.1.4 Stationary obstacle avoidance functions.

- **Disk obstacle avoidance:** For the *i*th swarm agent to avoid the *k*th static disk obstacle, the function
  $$\begin{array}{c} W_{ik}(𝐱)=\frac{1}{2}\left((x_{i}-{\text{ox}}_{k}{)}^{2}+(y_{i}-{\text{oy}}_{k}{)}^{2}-(r_{wc}+r_{k}{)}^{2}\right) \end{array}$$ (29)
  will be utilized.
- **Line obstacle avoidance:** To confine the swarm agents within designated boundaries, such as pavement edges, walls of buildings, or corridor walls, these boundaries are modeled as straight-line segments. Consistent with the Minimum Distance Technique (MDT) [29], the *i*th agent avoids the closest point on the *m*th line segment.

Let the *m*th line segment be defined by the endpoints $\left(e_{m1},f_{m1}\right)$ and $\left(e_{m2},f_{m2}\right)$. The coordinates of the closest point, $\left({\text{LX}}_{im},{\text{LY}}_{im}\right)$, are determined parametrically as:


$$\begin{array}{c} {\text{LX}}_{im}=e_{m1}+\lambda_{im}^{*}(e_{m2}-e_{m1}),\;{\text{LY}}_{im}=f_{m1}+\lambda_{im}^{*}(f_{m2}-f_{m1}). \end{array}$$ (30)

Using these coordinates, the following avoidance function is developed to ensure the *i*th agent maintains a safe distance from the *m*th line:


$$\begin{array}{c} B_{im}(𝐱)=\frac{1}{2}\left((x_{i}-{\text{LX}}_{im}{)}^{2}+(y_{i}-{\text{LY}}_{im}{)}^{2}-r_{wc}^{2}\right). \end{array}$$ (31)

The projection parameter $\lambda_{im}^{*}$ ensures the closest point lies strictly within the segment boundaries. It is defined as:


$$\begin{array}{c} \lambda_{im}^{*}=\left\{ \begin{array}{cc} 1, & \text{if }\lambda_{im}\geq 1\\ 0, & \text{if }\lambda_{im}\leq 0\\ \lambda_{im}, & \text{otherwise} \end{array}\right. \end{array}$$ (32)

where $\lambda_{im}$ is the calculated projection of the agent’s center onto the line vector and is given by


$$\begin{array}{c} \lambda_{im}=\frac{\left(e_{m2}-e_{m1})(x_{i}-e_{m1})+(f_{m2}-f_{m1})(y_{i}-f_{m1}\right)}{(e_{m2}-e_{m1}{)}^{2}+(f_{m2}-f_{m1}{)}^{2}}. \end{array}$$ (33)

#### 4.1.5 Centroid-based line obstacle avoidance.

To ensure the entire swarm avoids line obstacles, the function $H_{m}(𝐱)$ creates a repulsive potential based on the swarm’s centroid. The function


$$\begin{array}{c} H_{m}(𝐱)=\frac{1}{2}\left((\bar{x}-X_{m}{)}^{2}+(\bar{y}-Y_{m}{)}^{2}-(r_{wc}{)}^{2}\right) \end{array}$$ (34)

is used, where $\left(X_{m},Y_{m}\right)$ represents the closest point on the *m*th line obstacle to the swarm’s centroid. These coordinates are determined parametrically as


$$\begin{array}{c} X_{m}=e_{m1}+\lambda_{m}^{*}(e_{m2}-e_{m1}),\;Y_{m}=f_{m1}+\lambda_{m}^{*}(f_{m2}-f_{m1}). \end{array}$$ (35)

The projection parameter $\lambda_{m}^{*}$ ensures the closest point lies strictly within the segment boundaries. It is defined as:


$$\begin{array}{c} \lambda_{m}^{*}=\left\{ \begin{array}{cc} 1, & \text{if }\lambda_{m}\geq 1\\ 0, & \text{if }\lambda_{m}\leq 0\\ \lambda_{m}, & \text{otherwise} \end{array}\right. \end{array}$$ (36)

where $\lambda_{m}$ is the projection of the centroid onto the line vector and is given by


$$\begin{array}{c} \lambda_{m}=\frac{\left(e_{m2}-e_{m1})(\bar{x}-e_{m1})+(f_{m2}-f_{m1})(\bar{y}-f_{m1}\right)}{(e_{m2}-e_{m1}{)}^{2}+(f_{m2}-f_{m1}{)}^{2}}. \end{array}$$ (37)

#### 4.1.6 Artificial obstacle (Velocity Barrier) functions.

To enforce physical velocity limits, artificial obstacles are used. For a maximum angular wheel velocity of $v_{\max}$, which is assumed to be uniform for all Segways in the swarm, the barrier functions are:


$$\begin{array}{c} B_{R_{i}}(𝐱)=\frac{1}{2}(v_{\max}^{2}-v_{R_{i}}^{2})\;\text{and}\;B_{L_{i}}(𝐱)=\frac{1}{2}(v_{\max}^{2}-v_{L_{i}}^{2}). \end{array}$$ (38)

**Fig 4:**
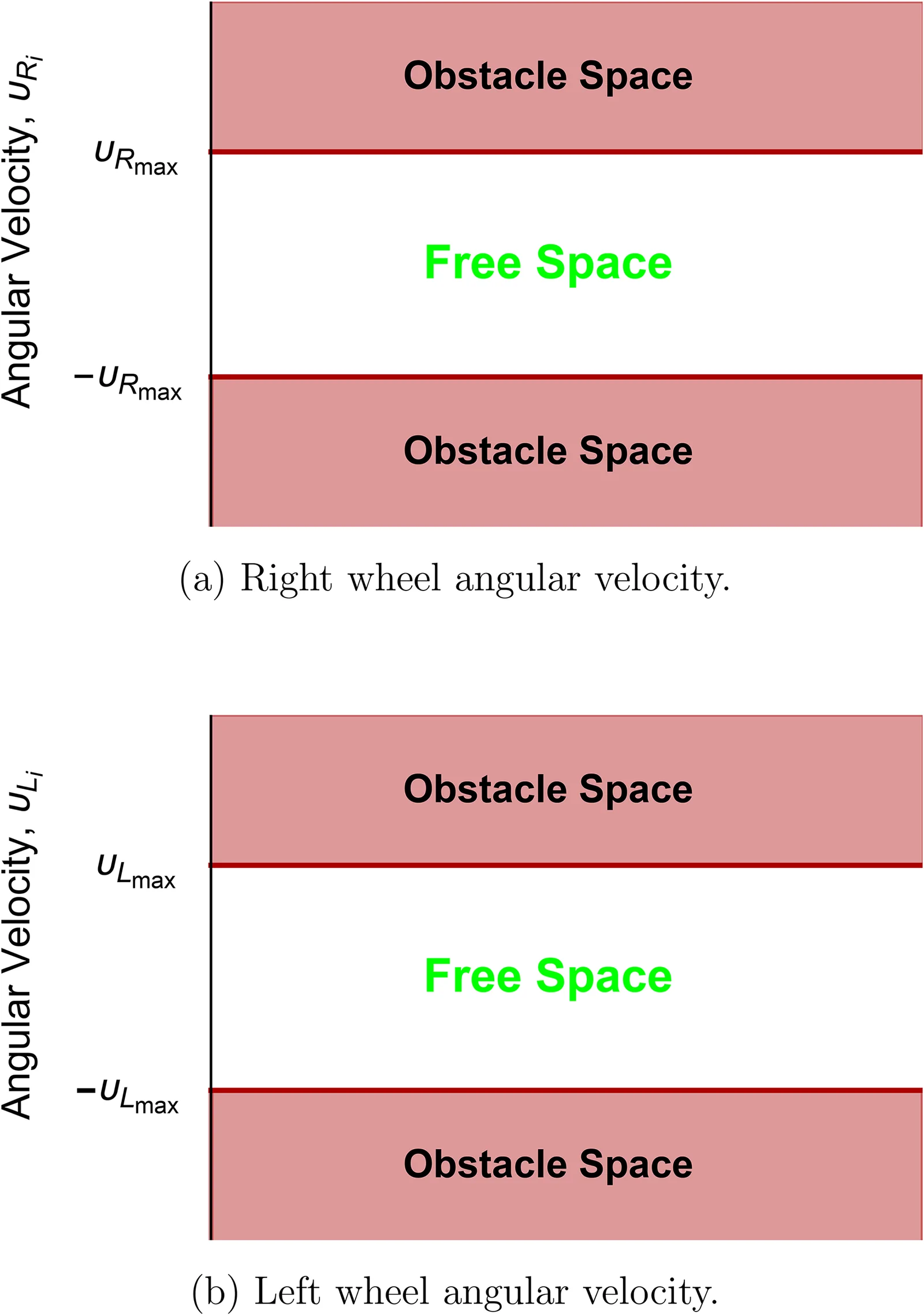
Visualization of the artificial velocity barriers. The central “Free Space” represents the safe operational range for each wheel’s angular velocity, while the shaded “Obstacle Space” represents prohibited speeds that the controller is designed to avoid.

These barrier functions translate the physical speed limits of the Segway’s motors into a mathematical construct that the LbCS can interpret as an obstacle. As visualized in Fig 4, this creates a “Free Space” of allowable angular velocities for the right and left wheels, bounded by a prohibited “Obstacle Space” representing speeds beyond the maximum limit, $v_{\max}$.

In the total potential function, these barriers are implemented as inverse functions (e.g., $1/B_{R_{i}}(𝐱)$). As a wheel’s angular velocity ($v_{R_{i}}$) approaches its maximum ($v_{\max}$), the value of $B_{R_{i}}(𝐱)$ approaches zero, causing the inverse term to grow towards infinity. This creates a steep “energy wall” that generates a strong repulsive force in the controller’s calculations. This force proactively commands deceleration, ensuring that angular velocity limits are never violated and that all control actions remain within the robot’s physical capabilities.

### 4.2 Dynamic obstacle avoidance and navigation functions

To model a realistic smart city environment, an asymmetric (one-way) avoidance scheme is implemented. The Segway swarm agents actively detect and avoid the dynamic obstacles. However, the dynamic obstacles that represent entities, such as pedestrians or manually driven vehicles, navigate toward their respective targets independently. They avoid static boundaries and each other, but operate with zero knowledge of the Segway swarm, meaning they do not yield to the autonomous robots.

#### 4.2.1 Swarm to dynamic obstacle avoidance.

For the *i*th Segway agent to actively avoid the *h*th dynamic obstacle in its path, the following repulsive avoidance function is incorporated into the swarm’s control architecture:


$$\begin{array}{c} N_{ih}(𝐱)=\frac{1}{2}\left((x_{i}-x_{d_{h}}{)}^{2}+(y_{i}-y_{d_{h}}{)}^{2}-(r_{wc}+r_{d_{h}}{)}^{2}\right) \end{array}$$ (39)

for i∈{1,...,n} and h∈{1,...,p}. As the dynamic obstacle approaches the Segway’s operational radius, this function approaches zero, generating a strong repulsive force that alters the Segway’s trajectory.

#### 4.2.2 Dynamic obstacle target attraction and auxiliary functions.

To ensure the convergence of the *h*th dynamic obstacle to its designated target, the following radically unbounded function is employed:


$$\begin{array}{c} V_{h}(𝐱)=\frac{1}{2}\left((x_{d_{h}}-\tau_{h_{1}}{)}^{2}+(y_{d_{h}}-\tau_{h_{2}}{)}^{2}+v_{d_{h}}^{2}+w_{d_{h}}^{2}\right). \end{array}$$ (40)

To guarantee that the nonlinear acceleration controllers of the *h*th dynamic obstacle vanish solely at its target equilibrium position, the corresponding auxiliary function is defined as:


$$\begin{array}{c} G_{d_{h}}(𝐱)=\frac{1}{2}\left((x_{d_{h}}-\tau_{h_{1}}{)}^{2}+(y_{d_{h}}-\tau_{h_{2}}{)}^{2}\right). \end{array}$$ (41)

#### 4.2.3 Dynamic obstacle collision avoidance functions.

The dynamic obstacles navigate the workspace independently, avoiding other dynamic entities, stationary disk obstacles, and stationary line boundaries.

- **Inter dynamic obstacle avoidance:** For the *h*th dynamic obstacle to avoid the *l*th dynamic obstacle, where h,l∈{1,...,p} and h≠l, the avoidance function is:
  $$\begin{array}{c} A_{d_{hl}}(𝐱)=\frac{1}{2}\left((x_{d_{h}}-x_{d_{l}}{)}^{2}+(y_{d_{h}}-y_{d_{l}}{)}^{2}-(r_{d_{h}}+r_{d_{l}}{)}^{2}\right). \end{array}$$ (42)
- **Stationary disk obstacle avoidance:** For the *h*th dynamic obstacle to avoid the *k*th stationary disk obstacle, for k∈{1,...,q}:
  $$\begin{array}{c} W_{d_{hk}}(𝐱)=\frac{1}{2}\left((x_{d_{h}}-o_{x_{k}}{)}^{2}+(y_{d_{h}}-o_{y_{k}}{)}^{2}-(r_{d_{h}}+r_{k}{)}^{2}\right). \end{array}$$ (43)
- **Stationary line obstacle avoidance:** For the *h*th dynamic obstacle to avoid the *m*th stationary line obstacle, for m∈{1,...,M}:
  $$\begin{array}{c} B_{d_{hm}}(𝐱)=\frac{1}{2}\left((x_{d_{h}}-{\text{LX}}_{hm}{)}^{2}+(y_{d_{h}}-{\text{LY}}_{hm}{)}^{2}-r_{d_{h}}^{2}\right). \end{array}$$ (44)

#### 4.2.4 Dynamic obstacle velocity constraints.

Translational velocity constraints for the dynamic obstacles are incorporated via artificial barrier functions. Let $v_{h_{\max}}$ and $w_{h_{\max}}$ denote the maximum allowable velocities. The avoidance functions are defined as:


$$\begin{array}{c} H_{1_{h}}(𝐱)=\frac{1}{2}\left(v_{h_{\max}}^{2}-v_{d_{h}}^{2}\right),\;H_{2_{h}}(𝐱)=\frac{1}{2}\left(w_{h_{\max}}^{2}-w_{d_{h}}^{2}\right). \end{array}$$ (45)

### 4.3 Total potential

A Lyapunov function, L(𝐱), combines the component functions with positive gains. The function is designed using inverse barrier functions for repulsion and auxiliary functions for scaling, ensuring the potential vanishes only at the desired equilibrium state. The Lyapunov function for the Segway swarm system and dynamic obstacle with an environment is:


$$\begin{array}{cc} L(𝐱)=\; & \alpha T(𝐱)+F(𝐱)(\sum_{i=1}^{n}(\gamma_{i}G_{i}(𝐱)+\sum_{\begin{array}{c} j=1\\ j\neq i \end{array}}^{n}\frac{\beta_{ij}}{A_{ij}(𝐱)}+\sum_{k=1}^{q}\frac{\mu_{ik}}{W_{ik}(𝐱)}\\ & +\sum_{m=1}^{M}\frac{g_{im}}{B_{im}(𝐱)}+\sum_{h=1}^{p}\frac{\omega_{ih}}{N_{ih}(𝐱)}+\frac{\Omega_{i}}{B_{L_{i}}(𝐱)}+\frac{{℧}_{i}}{B_{R_{i}}(𝐱)})+\sum_{m=1}^{M}\frac{h_{m}}{H_{m}(𝐱)})\\ & +\sum_{h=1}^{p}(V_{h}(𝐱)+G_{d_{h}}(𝐱)(\sum_{\begin{array}{c} l=1\\ l\neq h \end{array}}^{p}\frac{\alpha_{hl}}{A_{d_{hl}}(𝐱)}+\sum_{k=1}^{q}\frac{\eta_{hk}}{W_{d_{hk}}(𝐱)}\\ & +\sum_{m=1}^{M}\frac{{℘}_{hm}}{B_{d_{hm}}(𝐱)}+\frac{\xi_{h}}{H_{1_{h}}(𝐱)}+\frac{\zeta_{h}}{H_{2_{h}}(𝐱)})) \end{array}$$ (46)

where


$$\begin{array}{c} F(𝐱)=\frac{1}{2}\left((\bar{x}-\tau_{1}{)}^{2}+(\bar{y}-\tau_{2}{)}^{2}+\sum_{i=1}^{n}(v_{L_{i}}^{2}+v_{R_{i}}^{2})\right). \end{array}$$ (47)

is an auxiliary function ensuring all the repulsive potentials become zero only when the swarm centroid reaches its target, and the segway agents’ velocity reaches:

#### 4.3.1 Roles of control parameters.

The numerator terms in the total potential function L(𝐱) serve as tunable control gains that determine the relative influence of each attractive and repulsive component. Their physical interpretations are as follows:

- **Target convergence parameter (**α>0**):** This gain determines the strength of the attraction between the swarm’s centroid and the global target $\left(\tau_{1},\tau_{2}\right)$. A larger α results in faster convergence of the swarm centroid toward the destination.
- **Cohesion parameter (**$\gamma_{i}>0$**):** This parameter acts as a measurement of the strength of attraction between the *i*th agent and the swarm centroid. It governs the compactness of the formation; a larger $\gamma_{i}$ forces the agents to stay closer together, enhancing swarm cohesion.
- **Inter-agent coupling parameter (**$\beta_{ij}>0$**):** This gain represents the strength of the repulsive interaction between agent *i* and agent *j*. It scales the height of the repulsive potential barrier; a higher value creates a stronger repulsive force at greater distance, ensuring agents initiate avoidance maneuvers earlier to prevent collisions.
- **Obstacle avoidance parameters (**$\mu_{ik},g_{im},h_{m}>0$**):** These parameters scale the repulsion from static environments.
  - $\mu_{ik}$ determines the repulsion strength from the *k*th disk obstacle.
  - $g_{im}$ determines the repulsion strength for the *i*th agent from the *m*th line obstacle.
  - $h_{m}$ determines the repulsion strength for the swarm centroid from the *m*th line obstacle.

These gains function similarly to the obstacle avoidance parameters used in standard potential field methods, ensuring that the potential energy rises sufficiently high to prevent any agent from contacting an obstacle.

- **Velocity barrier gains (**$\Omega_{i},{℧}_{i}>0$**):** These parameters scale the artificial potentials associated with the wheel velocity limits. They ensure that as the wheel velocities $v_{L_{i}}$ or $v_{R_{i}}$ approach $v_{\max}$, the repulsive force becomes dominant, proactively decelerating the Segway to keep it within its physical capabilities.
- **Dynamic avoidance parameters (**$\omega_{ih},\alpha_{hl},\eta_{hk},{℘}_{hm}$**):** These gains dictate the sensitivity and repulsion strength for navigating the dynamic workspace. Specifically, $\omega_{ih}$ determines the strength of the avoidance maneuver executed by the *i*th Segway when encountering the *h*th dynamic obstacle. For the independent dynamic obstacles, $\alpha_{hl}$ governs inter-dynamic obstacle avoidance, while $\eta_{hk}$ and ${℘}_{hm}$ scale their repulsion from stationary disks and lines, respectively.
- **Dynamic velocity barrier gains (**$\xi_{h},\zeta_{h}>0$**):** Similar to the Segway constraints, these parameters scale the artificial potentials that actively enforce the translational velocity limits ($v_{h_{\max}},w_{h_{\max}}$) of the dynamic obstacles.

### 4.4 Controller design

The control inputs are derived to ensure the system satisfies the Lyapunov stability criterion $\dot{L}(𝐱)\leq 0$. The derivation commences with the time derivative of the Lyapunov function along the trajectories of the kinematic model defined in (9). Using the chain rule:


$$\begin{array}{cc} \dot{L}(𝐱) & =\sum_{i=1}^{n}\left(\frac{\partial L}{\partial x_{i}}{\dot{x}}_{i}+\frac{\partial L}{\partial y_{i}}{\dot{y}}_{i}+\frac{\partial L}{\partial \theta_{i}}{\dot{\theta }}_{i}+\frac{\partial L}{\partial v_{R_{i}}}a_{R_{i}}+\frac{\partial L}{\partial v_{L_{i}}}a_{L_{i}}\right)\\ & \;+\sum_{h=1}^{p}\left(\frac{\partial L}{\partial x_{d_{h}}}{\dot{x}}_{d_{h}}+\frac{\partial L}{\partial y_{d_{h}}}{\dot{y}}_{d_{h}}+\frac{\partial L}{\partial v_{d_{h}}}u_{1_{h}}+\frac{\partial L}{\partial w_{d_{h}}}u_{2_{h}}\right). \end{array}$$ (48)

Substituting the kinematic equations from (9) and grouping the terms by the respective velocity components yields:


$$\begin{array}{cc} \dot{L}(𝐱) & =\sum_{i=1}^{n}[v_{R_{i}}(\Psi_{R_{i}}+\frac{1}{v_{R_{i}}}\frac{\partial L}{\partial v_{R_{i}}}a_{R_{i}})+v_{L_{i}}(\Psi_{L_{i}}+\frac{1}{v_{L_{i}}}\frac{\partial L}{\partial v_{L_{i}}}a_{L_{i}})]\\ & \;+\sum_{h=1}^{p}[v_{d_{h}}(\frac{\partial L}{\partial x_{d_{h}}}+\frac{1}{v_{d_{h}}}\frac{\partial L}{\partial v_{d_{h}}}u_{1_{h}})+w_{d_{h}}(\frac{\partial L}{\partial y_{d_{h}}}+\frac{1}{w_{d_{h}}}\frac{\partial L}{\partial w_{d_{h}}}u_{2_{h}})] \end{array}$$ (49)

where $\Psi_{R_{i}}$ and $\Psi_{L_{i}}$ map the spatial partial derivative terms for the *i*th Segway agent:


$$\begin{array}{cc} \Psi_{R_{i}} & =c\frac{\partial L}{\partial \theta_{i}}+c(b\sin\theta_{i}+d\cos\theta_{i})\frac{\partial L}{\partial y_{i}}+c(b\cos\theta_{i}-d\sin\theta_{i})\frac{\partial L}{\partial x_{i}},\\ \Psi_{L_{i}} & =-c\frac{\partial L}{\partial \theta_{i}}+c(b\sin\theta_{i}-d\cos\theta_{i})\frac{\partial L}{\partial y_{i}}+c(b\cos\theta_{i}+d\sin\theta_{i})\frac{\partial L}{\partial x_{i}}. \end{array}$$ (50)

To enforce asymptotic stability for the augmented system, the analytically derived time derivative of the total potential is set equal to a negative semi-definite form. By introducing strictly positive convergence parameters $\sigma_{1_{i}},\sigma_{2_{i}}>0$ for the Segway agents, and $\delta_{1_{h}},\delta_{2_{h}}>0$ for the dynamic obstacles (h∈{1,...,p}), the target condition is established as:


$$\begin{array}{c} \dot{L}(𝐱)=-\sum_{i=1}^{n}\left(\sigma_{1_{i}}v_{R_{i}}^{2}+\sigma_{2_{i}}v_{L_{i}}^{2}\right)-\sum_{h=1}^{p}\left(\delta_{1_{h}}v_{d_{h}}^{2}+\delta_{2_{h}}w_{d_{h}}^{2}\right)\leq 0. \end{array}$$ (51)

By equating the velocity-grouped terms from the analytical expansion to this negative semi-definite target, the individual acceleration components can be isolated. For example, equating the right wheel velocity term for the *i*th Segway yields:


$$\begin{array}{c} \Psi_{R_{i}}+\frac{1}{v_{R_{i}}}\frac{\partial L}{\partial v_{R_{i}}}a_{R_{i}}=-\sigma_{1_{i}}v_{R_{i}}. \end{array}$$ (52)

Solving this algebraically for all control inputs results in the final decentralized nonlinear feedback control laws. The acceleration controllers governing the right and left wheels of the *i*th Segway agent are:


$$\begin{array}{c} a_{R_{i}}=-\left(\frac{v_{R_{i}}}{\frac{\partial L}{\partial v_{R_{i}}}}\right)(\Psi_{R_{i}}+\sigma_{1_{i}}v_{R_{i}}) \end{array}$$ (53)

$$\begin{array}{c} a_{L_{i}}=-\left(\frac{v_{L_{i}}}{\frac{\partial L}{\partial v_{L_{i}}}}\right)(\Psi_{L_{i}}+\sigma_{2_{i}}v_{L_{i}}) \end{array}$$ (54)

Concurrently, the translational acceleration controllers dictating the autonomous navigation of the *h*th dynamic obstacle are extracted as:


$$\begin{array}{c} u_{1_{h}}=-\left(\frac{v_{d_{h}}}{\frac{\partial L}{\partial v_{d_{h}}}}\right)\left(\frac{\partial L}{\partial x_{d_{h}}}+\delta_{1_{h}}v_{d_{h}}\right) \end{array}$$ (55)

$$\begin{array}{c} u_{2_{h}}=-\left(\frac{w_{d_{h}}}{\frac{\partial L}{\partial w_{d_{h}}}}\right)\left(\frac{\partial L}{\partial y_{d_{h}}}+\delta_{2_{h}}w_{d_{h}}\right) \end{array}$$ (56)

## 5 Simulation results

The simulation results presented in this section were generated using Wolfram Mathematica 14.3 software. The system of ordinary differential equations that defines the swarm’s dynamics, given in (9), was solved numerically using the Runge-Kutta 4th order (RK4) method. The positive control parameters (gains) used in the Lyapunov function and controllers were determined through an iterative tuning process (brute force) for each scenario to achieve effective and stable performance.

### 5.1 Simulation framework and units

The simulations were conducted within a dimensionless framework to ensure the generality of the results. All physical quantities are expressed in terms of Simulation Units (SU). For example, a distance is measured in spatial SU, and time is measured in temporal SU. A linear velocity reported by the simulation represents the movement of spatial SU per temporal SU.

This dimensionless approach allows the results to be mapped to a specific real-world physical scenario by defining a characteristic length, $L_{c}$ (e.g., in meters), and a characteristic time, $T_{c}$ (e.g., in seconds). A dimensionless linear velocity from the simulation, $v_{sim}$, can then be converted to a physical velocity, $v_{real}$, using the relationship:


$$\begin{array}{c} v_{real}=v_{sim}\frac{L_{c}}{T_{c}}. \end{array}$$ (57)

All angular quantities within the simulation, such as the orientation $\theta_{i}$, are expressed in radians. A simulated angular velocity, $\omega_{sim}$ (e.g., for $v_{L_{i}}$ or $v_{R_{i}}$), represents an angular displacement in radians per temporal SU. This can be converted to a physical angular velocity, $\omega_{real}$, by scaling with the inverse of the characteristic time:


$$\begin{array}{c} \omega_{real}=\frac{\omega_{sim}}{T_{c}}. \end{array}$$ (58)

This allows the generalized mathematical model to predict behavior in concrete situations with meaningful physical units (e.g., m/s or rad/s). Unless otherwise specified, all values reported in the following scenarios are in their direct Simulation Units.

### 5.2 Scenario 1: Consensus-based navigation in heterogeneous clutter with no Dynamic obstacles

This scenario evaluates the distributed control framework within a high-density environment designed to test cooperative maneuvering and formation control. A homogeneous swarm of *n* = 6 Segway agents is initialized with an arbitrary spatial distribution and tasked with performing a consensus-based rendezvous, resulting in a leader-follower formation.

The simulation workspace is populated with heterogeneous static constraints, comprising distinct geometric primitives: circular obstacles (pillars), convex polygons (triangles, hexagons, pentagons), and non-convex U-shaped barriers. This configuration compels the multi-agent system to deform its spatial arrangement to navigate narrow spaces before re-establishing formation integrity at the target. The specific initial states, obstacle geometric parameters, and consensus gains are detailed in Table 2.

**Table 2:** System parameters and environmental configuration for Scenario 1.

Parameter	Value / Description
**Swarm & Environment Configuration**	
Swarm Size (*n*)	6 Agents
Physical Dimensions (*b*, *d*)	0.5, 0.4 (SU)
Initial Velocities ($v_{R_{i_{0}}},v_{L_{i_{0}}}$)	(0,0) for all *i*
Initial Orientation ($\theta_{i_{0}}$)	Randomized
Constraint Type	Heterogeneous (Polygons, U-shapes, Disks)
Obstacle Population	21 Line barriers, 10 Disk obstacles (Randomized)
**Task Definition**	
Target Centroid ($\tau_{1},\tau_{2}$)	(165, 175)
Formation Topology	Linear target formation
**Control and Convergence Parameters**	
Attraction Gain (α)	0.5
Cohesion Shape Gain (δ)	0.1
Velocity Limits ($v_{\max}$)	1.0
Convergence Gains ($\sigma_{1_{i}},\sigma_{2_{i}}$)	(500, 500)
**Repulsion & Barrier Gains**	
$\gamma_{i},\beta_{ij},\mu_{ik},g_{im},h_{m}$	0.0002, 0.005, 0.005, 0.005, 0.005
$\Omega_{i},{℧}_{i}$	0.00001, 0.00001
*i*={1,2,...,*n*},*m*={1,2,...,*M*}	j={1,2,...,n},j≠i.

**Fig 5:**
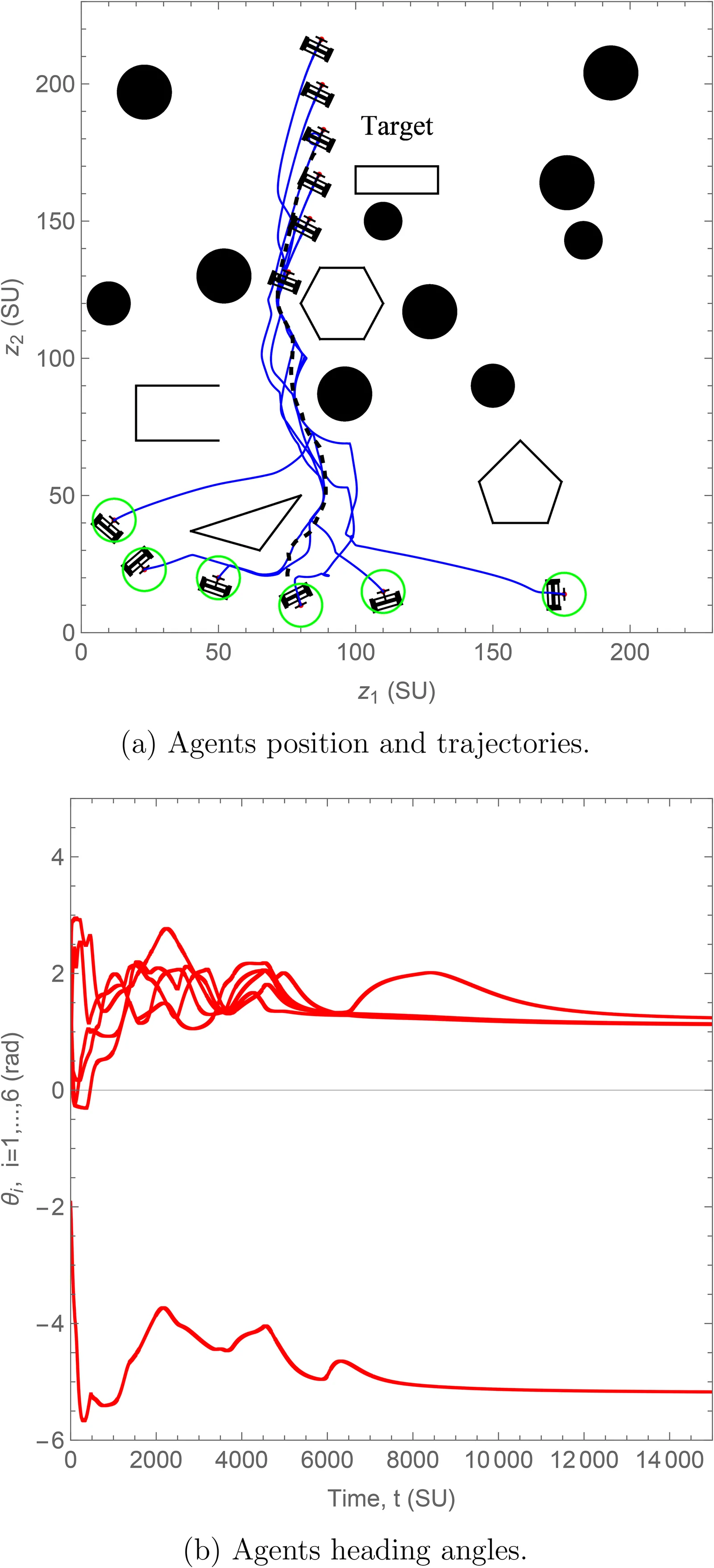
Swarm navigation performance in Scenario 1. (a) Complete spatial trajectories showing the transition from arbitrary initial poses (green circles) to the desired linear target formation. The blue lines track individual agents successfully executing collision-free maneuvers around heterogeneous static constraints (including disks, polygons, and a non-convex U-shaped barrier), while the dashed black line traces the swarm centroid’s path. (b) Time evolution of the heading angles $\theta_{i}(t)$. The significant oscillations observed during the transient phase (*t* < 8000 SU) quantitatively reflec*t* the active steering adjustments mandated by the non-holonomic kinematics for lateral obstacle avoidance. The subsequent asymptotic synchronization to steady-state values confirms robust orientation consensus as the swarm completely clears the clutter and aligns with the global target.

The simulation results are presented in Fig 5. Fig 5(a) illustrates the collision-free spatial evolution of the agents in the $\left(z_{1},z_{2}\right)$ plane. The agents exhibit emergent leader-follower behaviors to navigate the obstacle field, prioritizing collision avoidance during the transient phase before asymptotically converging to the desired linear formation.

Fig 5(b) presents the time history of the agent heading angles, $\theta_{i}(t)$. The oscillations observed in the transient phase (*t* < 8000) are attributed *t*o the active steering maneuvers required by the non-holonomic constraints of the Segway platform; lateral displacement for obstacle avoidance necessitates orientation changes. The evolution of these heading angles illustrates the emergent behavior resulting from the interplay of the continuous Lyapunov potential functions. The initial sharp spikes in $\theta_{i}$ correspond to the activation of the short-range repulsive potentials due to static obstacles and the strict enforcement of inter-agent separation. These repulsive potentials force the Segway agents into rapid steering maneuvers to prevent collisions. As time evolves and the swarm clears the cluttered region, long-range attraction dominates. The cohesion potential forces the agents to cluster around the centroid, ensuring centroid convergence. Furthermore, the heading angles asymptotically synchronize to a common bearing, indicating mutual alignment. This orientation consensus signifies that the agents have successfully transitioned from independent avoidance maneuvers to a unified, cooperative trajectory, ultimately achieving global target convergence and reaching their equilibrium point as a well-spaced, cohesive group.

**Fig 6:**
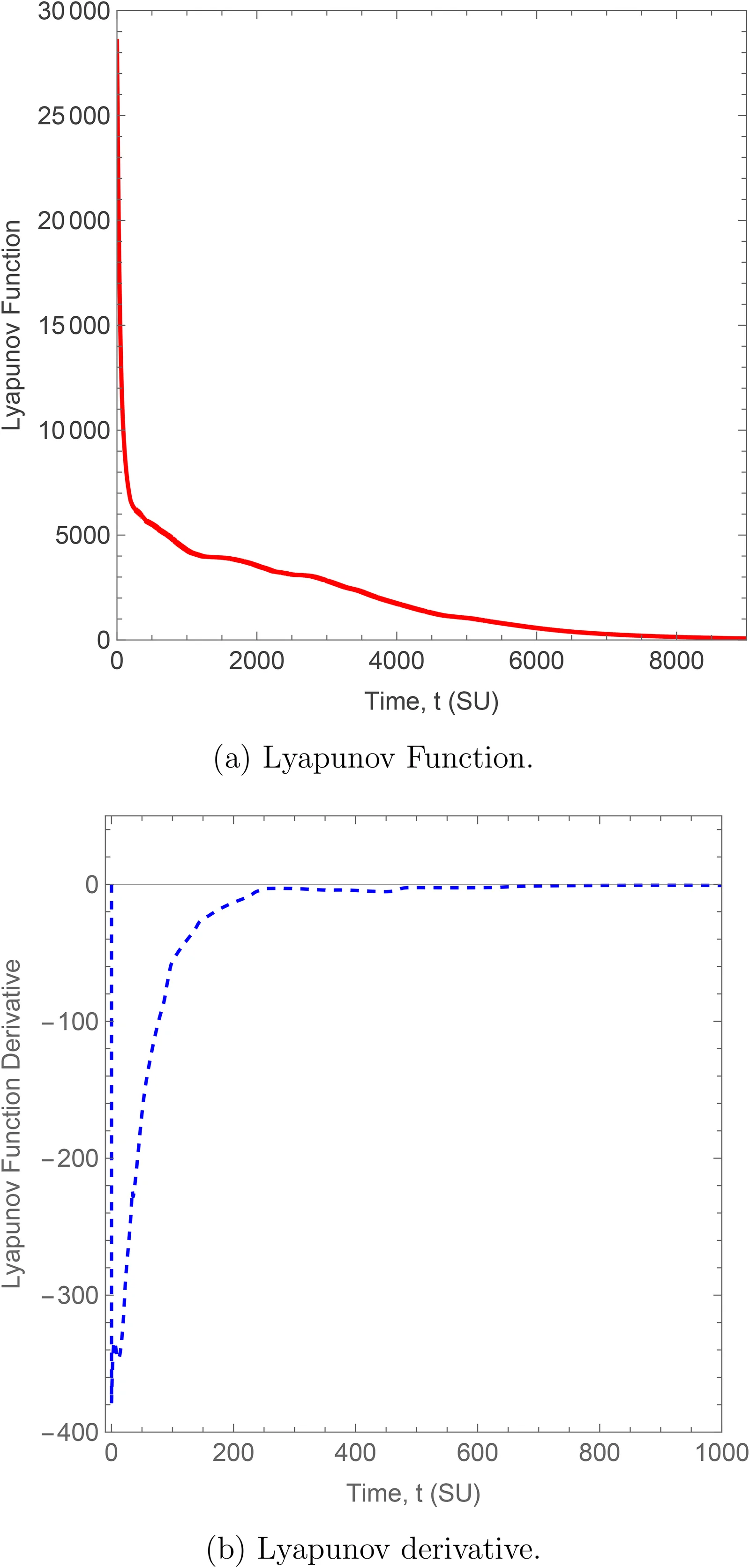
Quantitative stability verification for the multi-agent system in Scenario 1. (a) Evolution of the total Lyapunov potential L(𝐱). The system initializes with high potential energy (L≈30000) due to the distributed starting states, followed by a strict, monotonic decay. The asymptotic convergence to zero as t→10000 SU confirms the swarm reaches the global equilibrium without entrapment in local minima. (b) Time evolution of the Lyapunov derivative $\dot{L}(𝐱)$. The trajectory exhibits an immediate, sharp negative peak (≈−400) during the initial transient phase (*t* < 200 SU), reflecting the aggressive energy dissipation during early obstacle avoidance and formation assembly. Crucially, the curve remains strictly non-positive ($\dot{L}\leq 0$) throughout the entire simulation, satisfying asymptotic stability.

The robustness of the control framework is validated through the quantitative energy analysis presented in Fig 6. Fig 6(a) depicts the time evolution of the composite Lyapunov function, L(𝐱). Significantly, the decay remains strictly monotonic throughout the interaction with the heterogeneous constraints; while the obstacle-avoidance potentials generate local repulsive forces, they do not violate the energy-dissipation condition. The asymptotic convergence of L(𝐱) to zero confirms that the system reaches the desired equilibrium state without exhibiting oscillatory behavior. Fig 6(b) confirms the negative semi-definiteness of the Lyapunov derivative, $\dot{L}(𝐱)$. The trajectory remains strictly non-positive ($\dot{L}\leq 0$) throughout the simulation horizon. In accordance with LaSalle’s Invariance Principle, this guarantees graphically that the system states converge to the largest invariant set where $\dot{L}=0$ (the equilibrium manifold), proving that the obstacle avoidance terms do not induce limit cycles.

**Fig 7:**
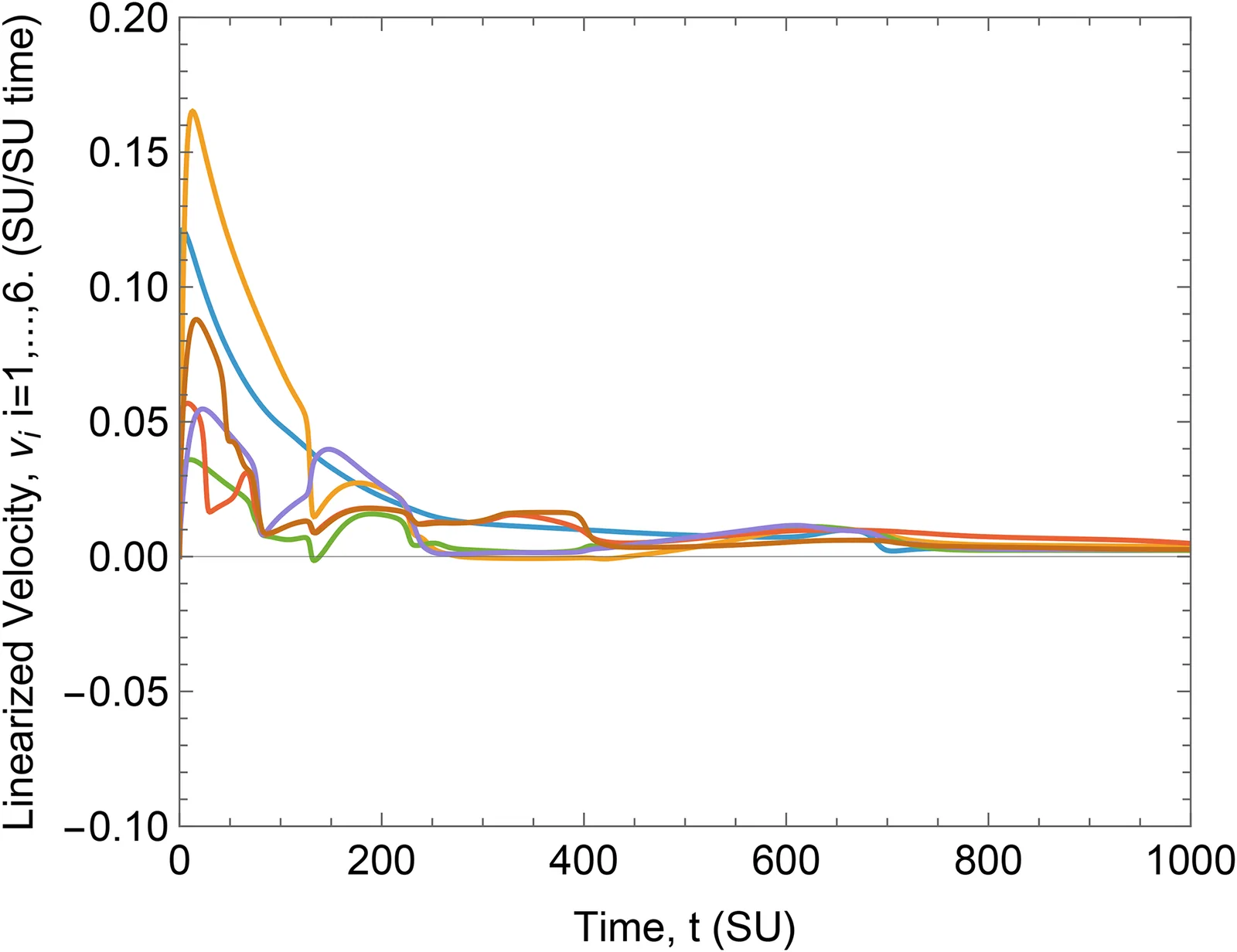
Time evolution of the linearized velocities $v_{i}(t)$ for the six agents in Scenario 1. At *t* = 0 SU, the agents initialize and rapidly accelera*t*e, with peak velocities reaching approximately 0.16 SU/SU time. The pronounced oscillations observed during the transient phase (*t* < 500 SU) quantitatively reflec*t* the active acceleration and braking maneuvers required to negotiate the heterogeneous obstacle field, including non-convex U-shaped barriers. As the swarm clears the clutter and establishes the leader-follower formation, the velocities smoothly dampen. The final asymptotic convergence of all agent velocities to zero ($v_{i}\to 0$) as t→1000 SU confirms the global asymptotic stability of the swarm at the target rendezvous configuration.

The time evolution of the linearized velocities $v_{i}(t)$ for the six swarm agents is depicted in Fig 7. The variations correspond to the active acceleration and deceleration maneuvers necessitated by the heterogeneous obstacle field, specifically the negotiation of non-convex U-shaped barriers and polygonal constraints. The sudden transient fluctuations in the velocity profiles illustrate the dynamic interplay between the attractive and repulsive potential functions. As agents approach static obstacles, a short-range repulsive force is generated, helping them navigate without collisions. The attraction potentials actively allow the forward momentum to ensure that the strict non-holonomic velocity bounds of the Segway platforms are not violated. This rapid velocity change is a direct consequence of the Lyapunov-based design, which actively enforces braking during tight avoidance maneuvers to guarantee rigorous collision avoidance before safely resuming steady-state convergence toward the target. As the agents clear the clutter and establish the leader-follower formation (approx. *t* > 10000 SU), the velocities monotonically decay. The asymp*t*otic convergence of all agent velocities to zero ($v_{i}\to 0$) as t→14000 confirms graphically that the swarm achieves a stable equilibrium at the target configuration.

### 5.3 Scenario 2: Robust navigation in semi-enclosed heterogeneous environments with no dynamic obstacles

Following the validation of the distributed control framework in open heterogeneous environments, Scenario 2 examines the system’s robustness and navigational efficacy within highly constrained, non-convex topologies. Specifically, this scenario evaluates the acceleration controller’s capability to resolve local minima problems inherent to semi-enclosed spaces. A swarm of *n* = 6 non-holonomic Segway agents is initialized within a warehouse-like enclosure, bounded by static linear constraints that form a geometric trap. To further increase the navigational complexity, the external environment is populated with a dense field of 13 static disk obstacles and 10 linear barriers. This configuration establishes a complex potential energy landscape, effectively segregating the agents from the target centroid at τ=(183,270) and necessitating advanced obstacle-avoidance and formation-deformation maneuvers. To minimize redundancy, only the parameters that differ from the baseline configuration in Scenario 1, specifically the initial positions, target coordinates, and velocity limits, are detailed in Table 3.

**Table 3:** Parameters for Scenario 2, listing only the values changed from Scenario 1.

Parameter	Value / Description
Constraint Type	Semi-enclosed Trap (13 Line Obs., 13 Disk Obs.)
Initial Positions ($(x_{i_{0}},y_{i_{0}}$)	(100,41), (130,14), (80,10), (10,57), (30,20), (50,23)
Target Centroid ($\tau_{1},\tau_{2}$)	(183, 270)
Cohesion Shape Gain (δ)	1.0
Velocity Limits ($v_{\max}$)	2.0

The primary objective is to verify that the acceleration controllers derived from the LbCS can successfully guide the agents out of the enclosure and converge towards the target. The framework must ensure a configuration free of local-minima traps while strictly maintaining safety with respect to inter-agent and obstacle collisions. The total potential function L(𝐱) for this constrained scenario exhibits strictly monotonic decay, similar to the graphical stability characteristics presented in Scenario 1.

**Fig 8:**
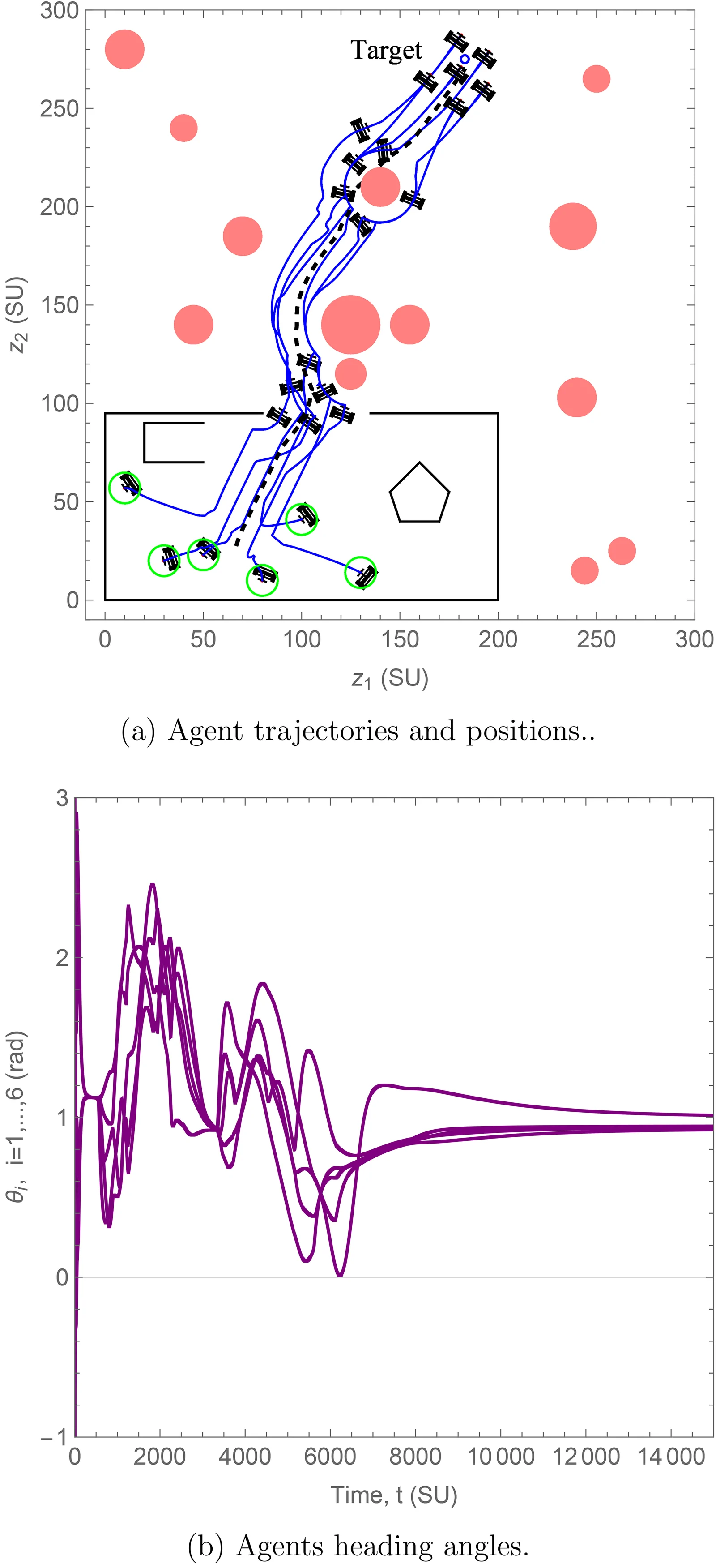
Simulation results for Scenario 2, illustrating swarm navigation through a constricted passage. (a) Agent trajectories and positional states. The position superimposed at time steps *t* = 0, 900, 5300, and 50000 SU to illustrate how the six-agent swarm avoids static obstacles and the enclosure boundaries. (b) Time evolution of the heading angles $\theta_{i}$ for all six agents. The initial fluctuations result from steering actions required to avoid the enclosure, while the subsequent convergence indicates alignment with the target.

The agent trajectories and positional states illustrated in Fig 8(a) demonstrate the swarm’s emergent capability to adapt its spatial topology to environmental constrictions. Specifically, a formation split-and-merge maneuver is observed as the swarm encounters the dense disk obstacle field at *t* = 5300 SU. The interplay between the inter-agent repulsion terms and the boundary potentials forces a spontaneous deformation of the swarm, allowing it to avoid the disk obstacle.

The time evolution of the agent heading angles, $\theta_{i}(t)$, presented in Fig 8(b), provides further insight into the control effort. The initial phase (*t* < 5000 SU) is characterized by high-frequency steering adjustmen*t*s. These oscillations correspond to the “negotiation phase,” in which agents actively balance repulsive forces. As the swarm clears the obstacle field and enters free space (*t* > 15000 SU), the heading angles asymptotically synchronize to a common angle, indica*t*ing that the swarm has achieved orientation consensus.

**Fig 9:**
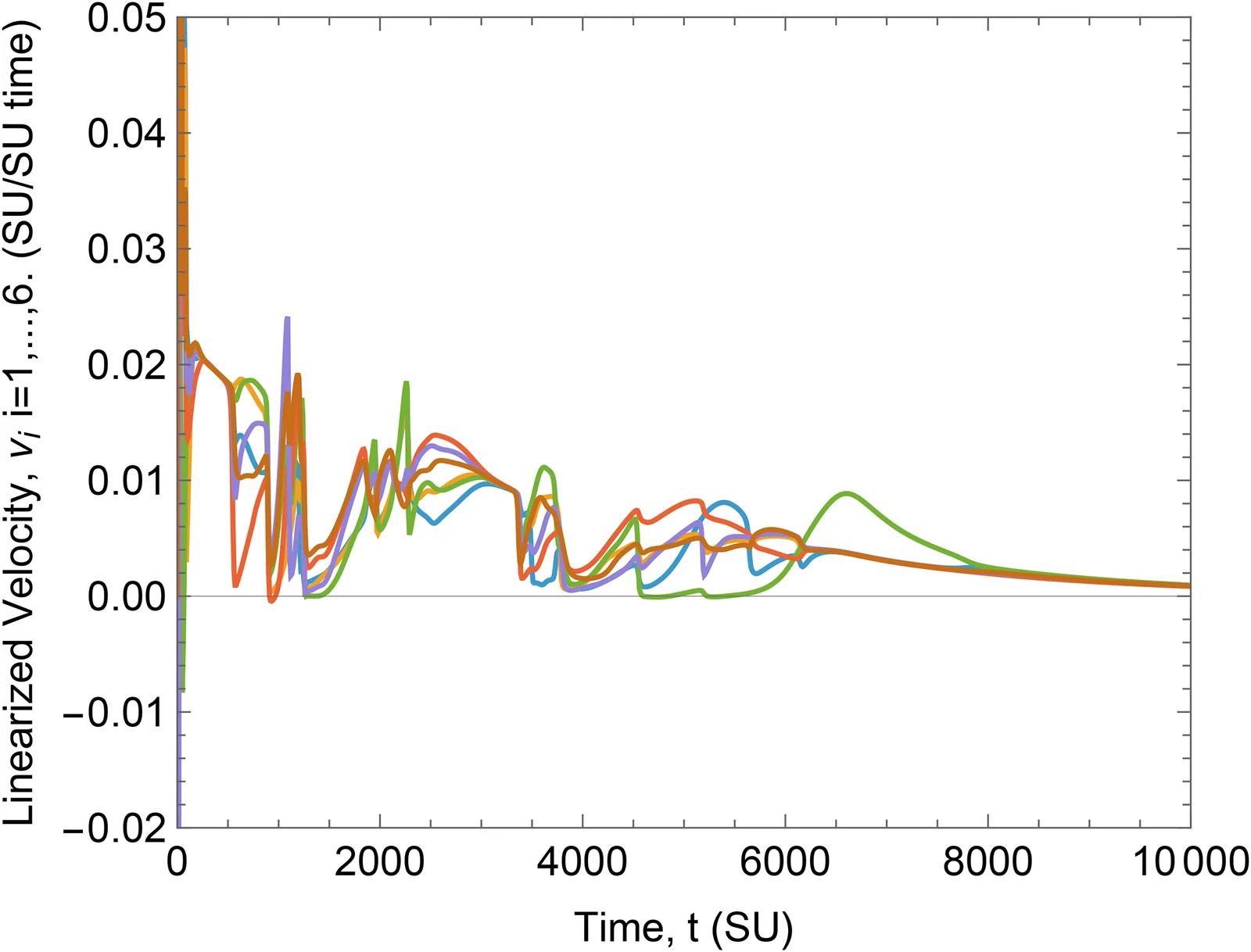
Time evolution of the linearized velocities $v_{i}(t)$ for the six agents in Scenario 2. At *t* = 0 SU, the agents initialize from res*t* and exhibit sharp acceleration spikes (reaching up to approximately 0.05 SU) as they react to the repulsive potentials of the semi-enclosed trap. The phase of high-amplitude modulations (*t* < 6000 SU) directly corresponds to the aggressive braking and accelera*t*ion maneuvers necessary to negotiate the dense field of 13 disk obstacles outside the trap. Once the swarm clears these constraints (*t* > 7000 SU), the velocities exhibit a smooth, mono*t*onic decay. The final asymptotic convergence to zero ($v_{i}\to 0$) as t→10000 SU confirms graphically that the system achieves global stability at the designated target configuration.

Finally, the stability of the system is corroborated by the time evolution of the linearized velocities, $v_{i}(t)$, as depicted in Fig 9. The initial phase (*t* < 6000 SU) is characterized by rapid, high-amplitude modulations. These *t*ransients correspond to the aggressive acceleration and braking maneuvers required to resolve the conflicting potential fields within the trap and the subsequent obstacle field. As the swarm agents navigate through the narrow enclosure exit, both inter-agent separation and repulsive potentials are effective due to static obstacles. The sharp dips in the velocity profile occur because of these steep, only momentarily present, repulsive potentials. The vanishing velocity profile ($\lim_{t\to \infty }v_{i}(t)=0$) confirms graphically that the system achieves a stable equilibrium at the desired configuration without exhibiting limit cycle behavior or residual oscillations.

### 5.4 Scenario 3: Distributed assembly and corridor navigation with no dynamic obstacles with no dynamic obstacles

**Fig 10:**
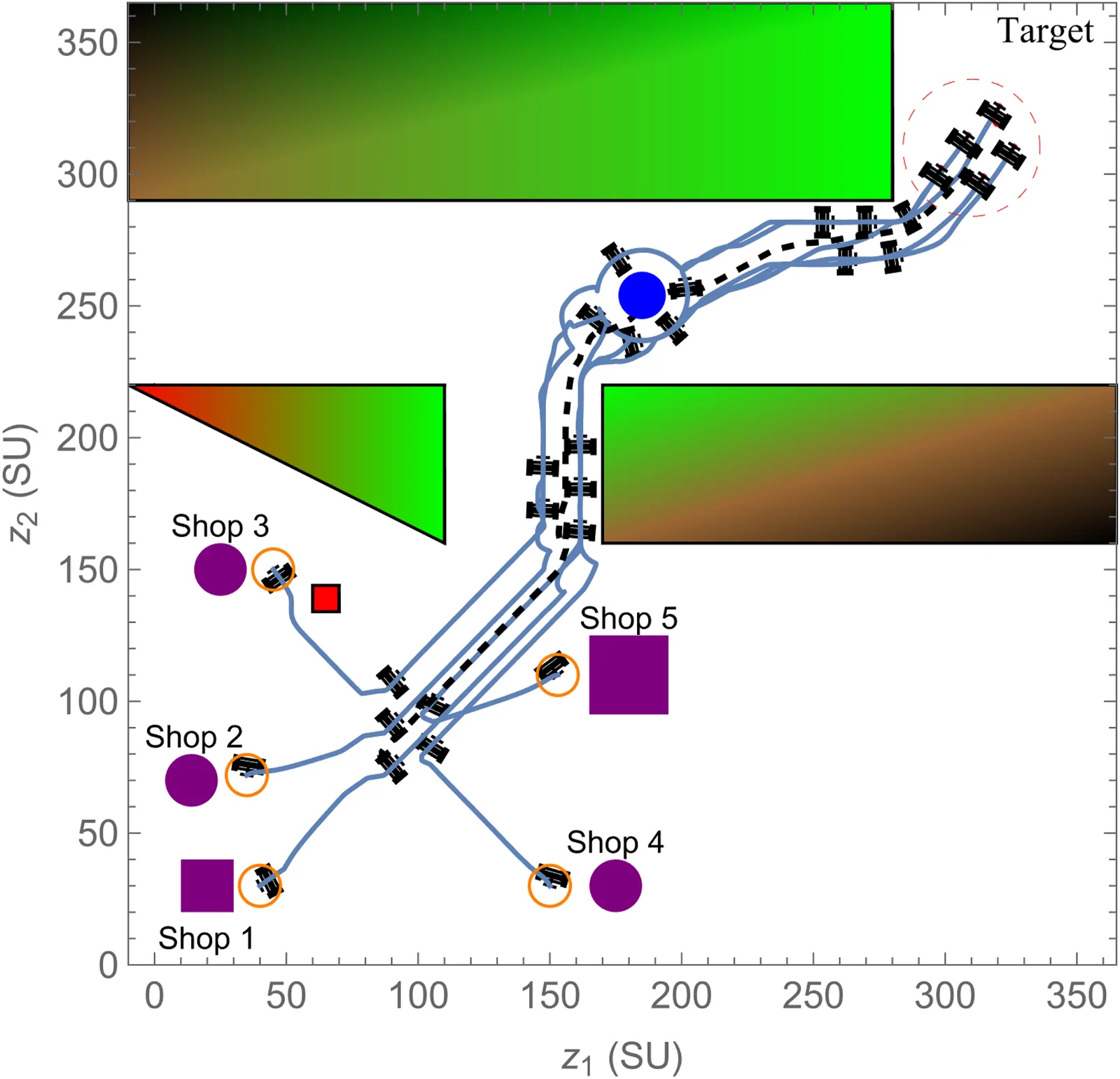
A sequence of snapshots illustrating the multi-agent rendezvous and corridor navigation task in Scenario 3. At *t* = 0, SU, the five agents are distributed across distinct nodes (Shops 1–5, indicated by orange circles). Driven by cohesion potential, the agents rapidly aggregate into a cohesive swarm (*t* = 120 SU) before entering the narrow corridor. A critical key trajectory feature occurs around *t* = 3600 SU, where the swarm executes a smooth split-and-merge maneuver to bypass the central blue disk obstacle. The sequence concludes with the successful asymptotic convergence of the entire formation at the designated target location (dashed red circle) by *t* = 15000 SU. The dashed black line traces the collision-free trajectory of the swarm’s centroid.

Scenario 3 examines the framework’s versatility through a distributed rendezvous problem, comparable to a decentralized logistics operation in which agents are dispatched from spatially distinct nodes (Shops 1–5) to a single customer location. The objective is to evaluate the effectiveness of the acceleration-based controllers in a dense environment. Fig 10 illustrates the Segway positions at six distinct time steps (*t* = 0, 120, 1200, 3600, 6000, 15000 SU), with the simulation parameters detailed in Table 4.

**Table 4:** System parameters and environmental configuration for Scenario 3.

Parameter	Value / Description
**Topological Configuration**	
Initial Positions ($(x_{i_{0}},y_{i_{0}}$)	(40,30),(150,30),(35,72),(45,72),(153,110)
Target Centroid (τ)	(310, 320)
Constraint Type	Heterogeneous obs. with narrow corridor
**Control Gains**	
Attraction Gain (α)	0.5
Cohesion Shape Gain (δ)	1.0
Velocity Limits ($v_{\max}$)	2.0
Convergence Gains ($\sigma_{1},\sigma_{2}$)	(500, 500)
Repulsion Gains ($\mu_{ik}$)	0.005
	*i*={1,2,...,*n*},*k*={1,2,...,*q*}

The system dynamics exhibit a distinct two-phase kinematic evolution driven by the superposition of the potential functions. In the initial phase (t≈120 SU), the formation cohesion term dominates the control effort. The aggregation observed in Fig 10 illustrates the complete interplay of the multiple attractive and repulsive potential functions comprising the Lyapunov formulation. The system is driven by a dual-attraction mechanism: the cohesion potential ($\gamma_{i}G_{i}(𝐱)$) pulls individual agents inward to ensure rapid centroid convergence, while the global attraction potential (αT(𝐱)) continuously pulls this swarm centroid toward the final destination. Simultaneously, various repulsive potentials remain continuously active. Short-range inter-agent separation potentials strictly prevent internal collisions as agents densely cluster, while the swarm safely navigates around static obstacles due to the interplay of repulsive potential functions.

Subsequently, the swarm navigates the corridor as a unified entity. Upon encountering the disk obstacle, the system exhibits a split-and-merge maneuver. The interaction between the obstacle-repulsive gradient and the inter-agent potentials forces a separation of the agent formation to avoid the disk obstacle. Following obstacle clearance, the formation effectively regains cohesion and asymptotically converges to the equilibrium, validating the system’s capability to execute complex coordination tasks in structured environments.

**Fig 11:**
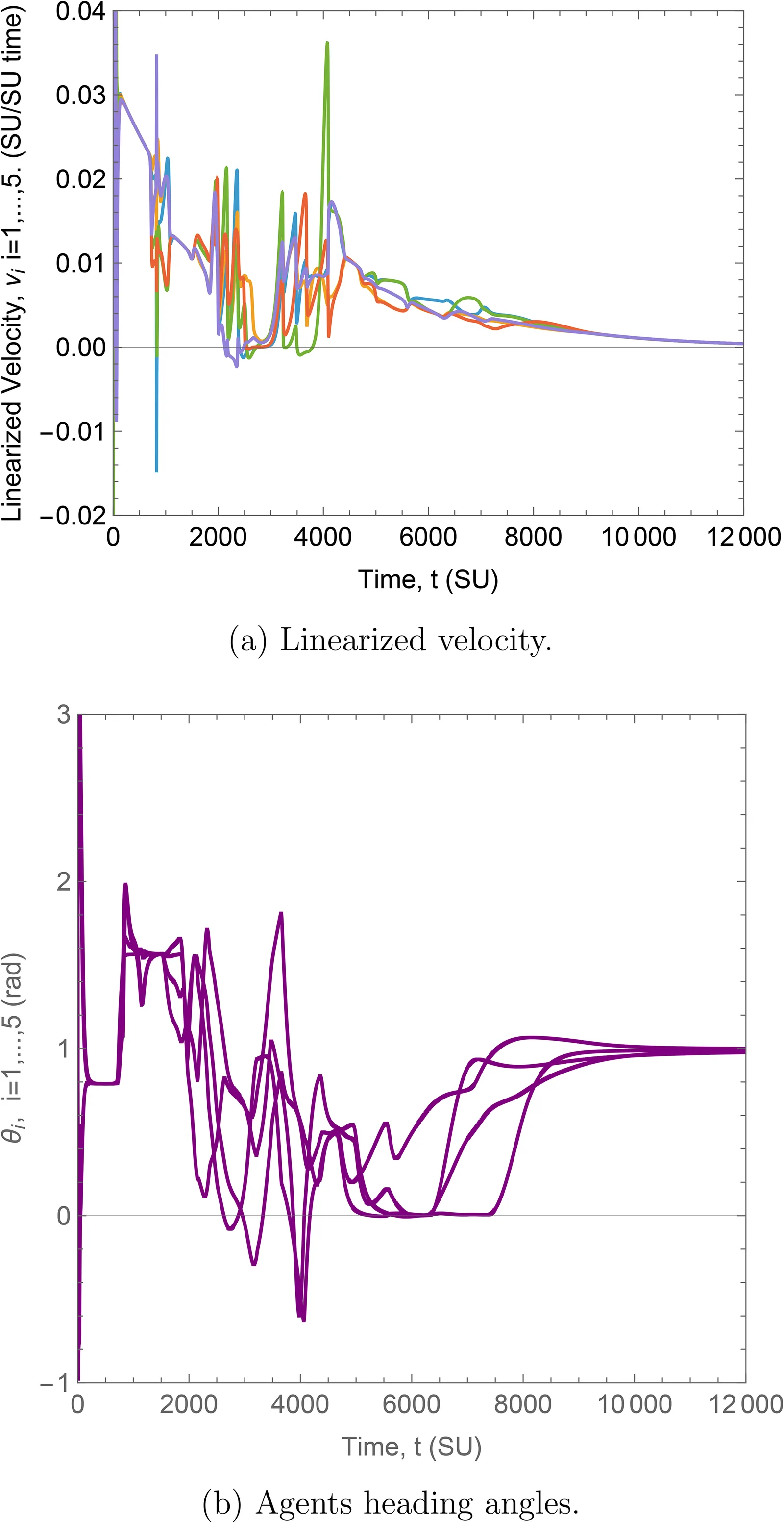
Control effort and state evolution for the five-agent swarm in Scenario 3. (a) The linearized velocities $v_{i}(t)$ of the agents. The initial transients (*t* < 6000 SU) correspond to the acceleration required for forma*t*ion assembly and the split-and-merge maneuver around the disk obstacle. The subsequent asymptotic decay to zero ($v_{i}\to 0$) confirms the system’s global stability at the target configuration. (b) Time evolution of the heading angles $\theta_{i}(t)$. The significant oscillations observed during the interval t∈[0,8000] SU reflect the active steering adjustments necessary to negotiate the corridor constraints and the disk obstacle. The final convergence to a common steady-state value indicates that orientation consensus is achieved as the swarm stabilizes in the safety zone.

The stability of the convergence is corroborated by the linearized velocity profiles $v_{i}(t)$ shown in Fig 11(a). The velocities exhibit a smooth, monotonic decay driven by the dissipative damping terms ($\Delta_{i}=500$). However, the transient spikes in both velocity and heading angle observed between *t* = 2000 and *t* = 6000 SU directly correlate to the split-and-merge maneuver. During this phase, *t*he gradien*t* of the disk obstacle’s repulsive potential forces a lateral divergence, which mathematically necessitates a temporary increase in linear velocity and a sharp deviation in heading to satisfy the under-actuated kinematic constraints before the formation can reunite. The asymptotic convergence to zero ($v_{i}\to 0$ as t→∞) confirms graphically that the system possesses global asymptotic stability at the target configuration.

The system’s dynamic response is further quantified by the temporal evolution of the agent states. Fig 11(b) presents the heading angles $\theta_{i}(t)$. As the swarm exits the corridor constraints, the heading angles asymptotically synchronize to a common steady-state value, confirming that orientation consensus is robust.

### 5.5 Scenario 4: Swarm navigation in a dynamic environment

To validate the framework’s safety in active urban environments, Scenario 4 evaluates the swarm’s performance when encountering three independent moving entities ($DO_{1},DO_{2},DO_{3}$). In this asymmetric avoidance task, a swarm of *n* = 15 Segway agents initializes at the top of the workspace and navigates toward a global target at τ=(50,50). The agents must avoid a static environment comprising 7 disks and 11 line barriers, while simultaneously avoiding inter-agent and dynamic obstacle collision.

These dynamic obstacles autonomously navigate toward their respective targets while actively avoiding the static environmental constraints. A one-way avoidance scheme has been used, in which swarm agents avoid only the dynamic obstacles. Evaluating this asymmetric formulation is highly relevant for validating real-world urban scenarios. First, it accurately models encounters with uncooperative or unaware entities commonly found in smart cities, such as distracted pedestrians, manually driven legacy vehicles, or animals. Second, it serves as a rigorous worst-case stress test for the proposed LbCS framework; by forcing the swarm to shoulder the entire physical burden of collision avoidance, it demonstrates the controller’s absolute safety guarantees without relying on the cooperative maneuvering of external agents. Finally, it reflects practical operational hierarchies where the swarm must actively yield the right-of-way to higher-priority or heavily constrained dynamic entities. The swarm possesses complete real-time state information of all entities within the workspace. The Segway agents rely entirely on the dynamic repulsive potential $N_{ih}(𝐱)$ to adjust their trajectories and avoid collisions with these non-reactive moving obstacles. Table 5 summarizes the configuration, environmental constraints, and specific control gains utilized for the multi-agent system in Scenario 4.

**Table 5:** System parameters and environmental configuration for Scenario 4.

Parameter	Value / Description
**Topological Configuration**	
Initial Positions ($\left(x_{i_{0}},y_{i_{0}}\right)$)	Randomized (15 Agents)
Target Centroid (τ)	(50, 50)
Constraint Type	Randomized 7 Disks, 11 Lines,
	3 Dynamic Obstacles
**Control Gains**	
Attraction Gain (α)	1.0
Cohesion Shape Gain (δ)	0.50
Velocity Limits ($v_{\max}$)	1.0
Segway Convergence Gains ($\sigma_{1_{i}},\sigma_{2_{i}}$)	(500, 500)
Dynamic Obs. Convergence Gain ($\delta_{1_{h}},\delta_{2_{h}}$)	(200,200)
Repulsion Gains ($\mu_{ik}$)	0.5
Dynamic Repulsion Gain ($\omega_{ih}$)	0.008
	i={1,2,...,n},h={1,2,...,p}
Dynamic Velocity Limits ($v_{d_{\max}}$)	1.2

**Fig 12:**
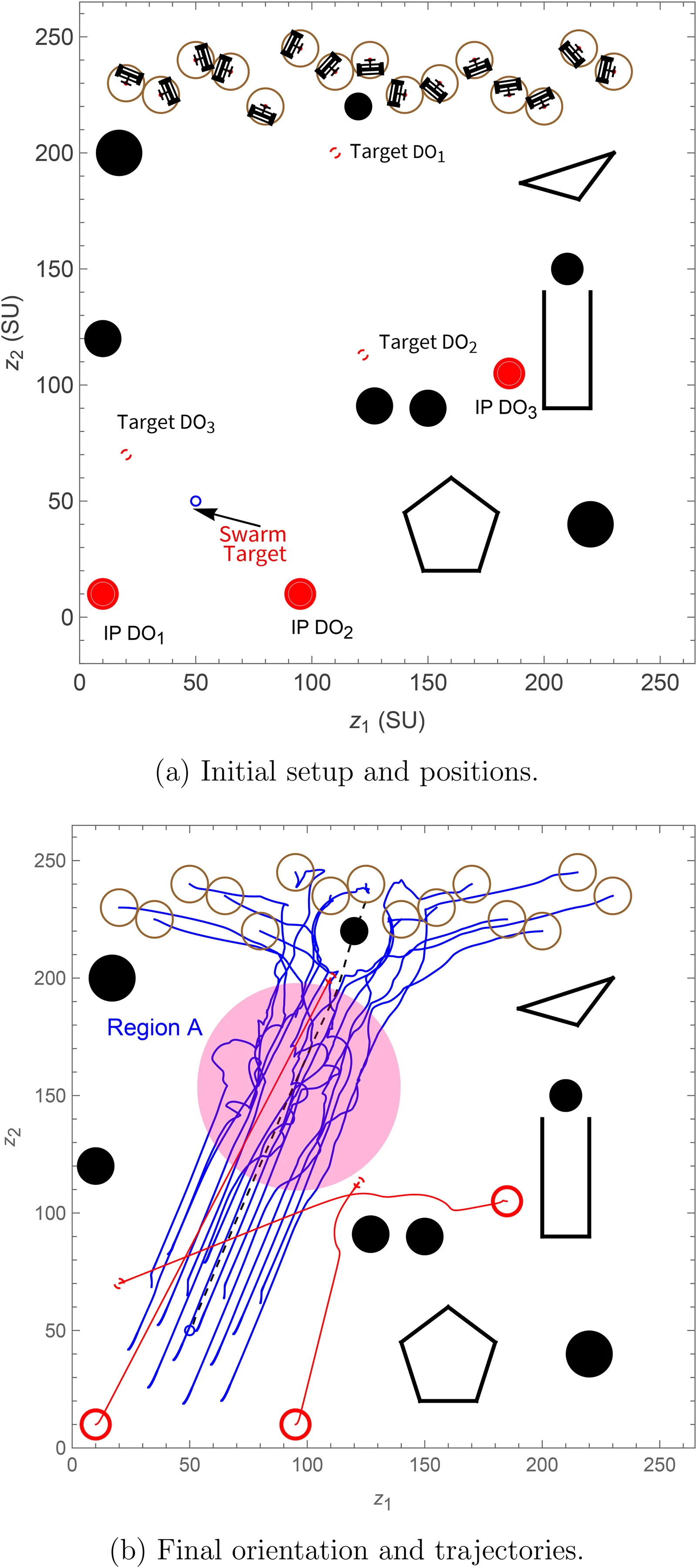
Global workspace configuration and spatial evolution for Scenario 4. (a) The initial spatial distribution of the *n* = 15 Segway agents alongside the three independent dynamic obstacles ($DO_{1},DO_{2},DO_{3}$) and their respective designated targets. (b) The complete trajectory trace of the multi-agent system. The blue curves represent the collision-free paths of the Segway agents, illustrating their successful navigation through heterogeneous static constraints (disk and line barriers) and their active evasion of the dynamic obstacles (red trajectories) prior to converging to global target equilibrium τ=(50,50).

Fig 12(a) illustrates the initial setup of the workspace, detailing the initial positions and respective targets for both the Segway swarm and the three dynamic obstacles. Fig 12(b) presents the complete spatial evolution of the system. The blue curves represent the trajectories of the swarm agents, confirming that the swarm successfully avoids the static constraints and converges at the global target while maintaining a strict safety distance from the dynamic obstacles $DO_{1},DO_{2},$ and *DO*_3_. The most critical interaction occurs in Region A, where the swarm’s planned trajectory intersects directly with the first dynamic obstacle (*DO*_1_). As observed in the spatial evolution in Fig 12(b), the proximity of this moving entity triggers the dynamic repulsive potential, forcing the swarm into a rapid local deformation to ensure collision avoidance before resuming its path to the target.

**Fig 13:**
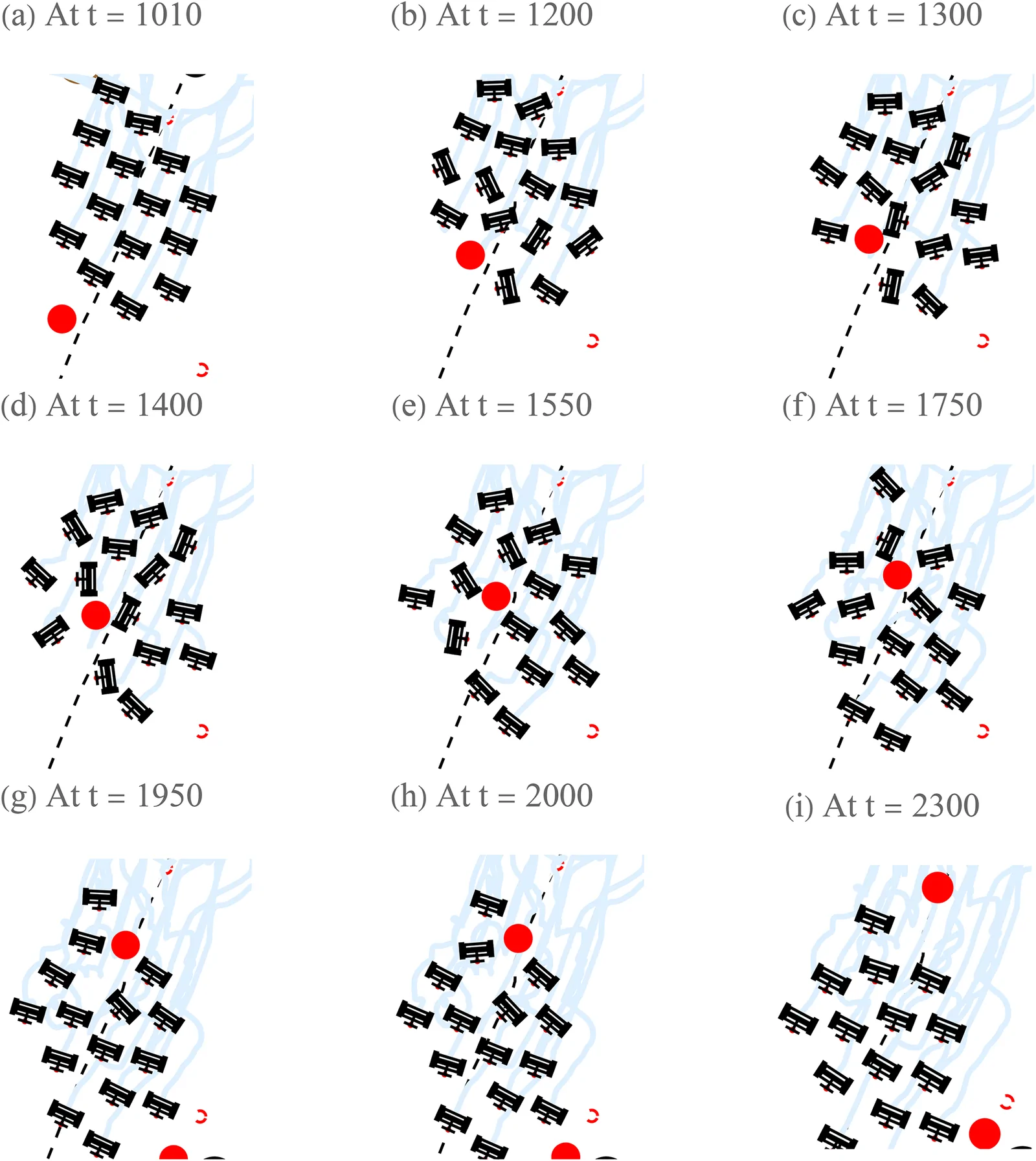
Snapshot sequence detailing the real-time dynamic avoidance maneuver in Region A. (a)-(c) At *t* = 1010 to 1300 SU, the 15-agent swarm detects the impending collision with the independent dynamic obstacle (*DO*_1_, red circle) approaching along the dashed trajectory. (d)-(f) Between *t* = 1400 and *t* = 1750 SU, the dynamic repulsive potential $N_{ih}(𝐱)$ dominates the control effort, forcing the agents to execute a rapid split-and-merge maneuver. The formation actively deforms, creating a safe passage corridor to prioritize collision avoidance over global cohesion. (g)-(i) Once *DO*_1_ clears the interaction zone (*t* > 1950 SU), the cohesion potential $\gamma_{i}G_{i}(𝐱)$ reasser*t*s control. By *t* = 2300 SU, the agents have successfully reconstituted their rigid formation and resumed their synchronized *t*rajectory toward the global target.

The avoidance maneuver within Region A is detailed via snapshots in Fig 13. As *DO*_1_ approaches the swarm’s centroid between *t* = 1010 and *t* = 1300, the repulsive potential $N_{ih}(𝐱)$ forces the leading agents to widen their formation. By *t* = 1550, the swarm actively splits *t*o create a safe passage for *DO*_1_. This split-and-merge reaction demonstrates that local repulsive functions momentarily override the global cohesion gain to prioritize collision avoidance. Once *DO*_1_ clears the interaction zone at *t* = 1950, the cohesion potential $\gamma_{i}G_{i}(𝐱)$ forces *t*he agents back into a rigid cluster. The swarm recovers its formation completely by *t* = 2300 and resumes its synchronized approach towards the target.

**Fig 14:**
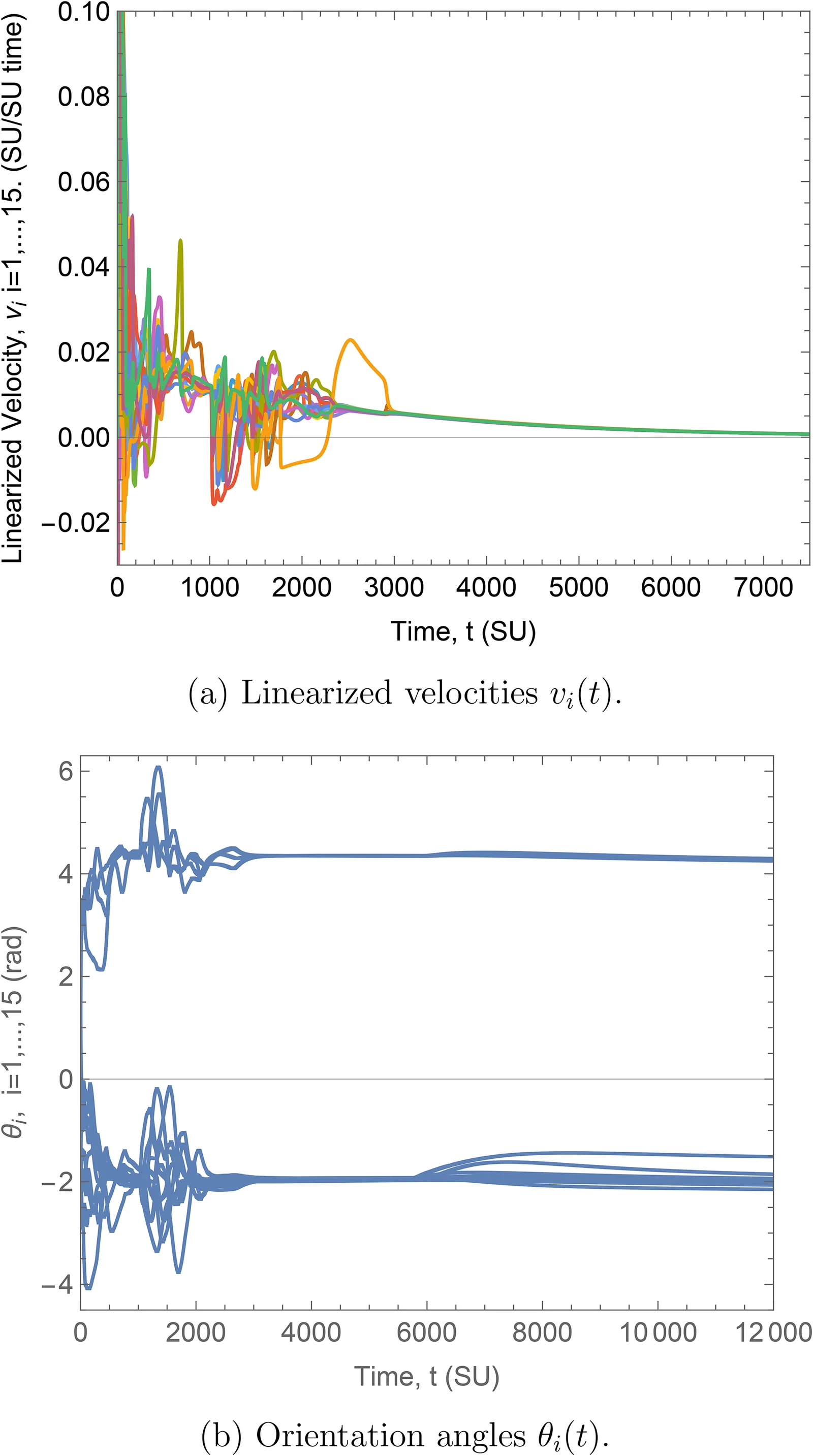
Kinematic profiles for the 15-agent Segway swarm in Scenario 4. (a) Time evolution of the linearized velocities $v_{i}(t)$. The pronounced transient fluctuations observed between t≈0 and t≈2500 SU directly correspond to the aggressive braking and acceleration maneuvers required to evade the independent dynamic obstacles. The subsequent asymptotic decay to zero ($v_{i}\to 0$ as t→7000 SU) confirms the system’s ultimate stabilization at the global equilibrium point. (b) Time evolution of the orientation angles $\theta_{i}(t)$. The sharp steering oscillations during the transient phase (*t* < 3000 SU) reflect the active heading adjustments necessary for the local split-and-merge avoidance maneuvers. Once the dynamic threats are cleared, the orientation angles asymptotically converge to their steady-state values, indicating orientation consensus and forma*t*ion integrity at the target.

The kinematic responses of the agents are quantified in Fig 14. The linearized velocity profile Fig 14(a) displays transient fluctuations corresponding directly to the active braking and acceleration required during the dynamic avoidance maneuvers. Specifically, the sharp change in velocity between t≈0 and t≈2500 SU is mathematically forced by the time-varying dynamic repulsion term $\frac{\omega_{ih}}{N_{ih}(𝐱)}$. As the dynamic obstacle *DO*_1_ approaches, the controllers aggressively brake and laterally disperse the swarm until the dynamic threat has passed. Simultaneously, strict inter-agent separation and repulsive potentials from static obstacles are actively enforced to prevent collisions. Once the dynamic threat passes, the dual-attraction mechanism reasserts dominance. The cohesion potential drives rapid centroid convergence to reform the formation, while the target convergence potential continues to pull the swarm toward the destination. Ultimately, the agents achieve mutual alignment and resume a synchronized, well-spaced trajectory toward the equilibrium point. Over time, these velocities strictly decay to zero, confirming asymptotic convergence at the equilibrium point. Similarly, the orientation angles $\theta_{i}$ Fig 14(b) stabilize after the collision avoidance phase, indicating that the entire multi-agent system achieves its desired final heading.

## 6 Discussion

Cooperative navigation capabilities are critical for smart city environments, where high-density traffic and shared infrastructure necessitate self-organizing systems to maximize operational safety. This study presented and validated a unified distributed control framework for a swarm of non-holonomic Segway robots. The effectiveness of the proposed controllers was rigorously evaluated across four progressively complex simulation scenarios, providing a proof of concept for the control design. The results confirm that the acceleration-level controllers provide a robust solution for multi-agent coordination in constrained, heterogeneous environments.

The system’s adaptability is evidenced by its consistent performance across diverse topological challenges:

1. **Baseline Navigation (Scenario 1):** The swarm successfully negotiated open fields populated with heterogeneous clutter, demonstrating fundamental leader-follower capabilities (Fig 5a).
2. **Trap Escape (Scenario 2):** The system exhibited *formation plasticity*, spontaneously reorganizing from a cluster into a linear stream to escape a non-convex semi-enclosed trap (Fig 8).
3. **Distributed Assembly (Scenario 3):** In a decentralized rendezvous task, the controller successfully aggregated spatially distributed agents into a cohesive unit and guided them through a constrained corridor, validating the framework’s stability (Fig 10).
4. **Dynamic Avoidance (Scenario 4):** The swarm successfully navigated a constrained workspace while actively adjusting its formation to avoid independent, non-reactive moving entities, proving the framework’s viability in time-varying environments (Fig 12).

### 6.1 Limitations and future work

While the simulation results demonstrate the theoretical robustness of the proposed framework, several limitations inherent to the study design must be acknowledged. These constraints define the trajectory for future research.

#### 6.1.1 Algorithmic and methodological constraints.

- **Dynamic state estimation:** While the framework successfully executes dynamic obstacle avoidance, the current mathematical model assumes the Segway agents possess perfect, real-time state information regarding the positions and velocities of moving entities. In real urban deployments, predicting the intent of unpredictable actors (e.g., pedestrians) introduces significant uncertainty, requiring high-quality sensors and robust noise handling. Future work will integrate predictive estimators to handle uncertain dynamic states.
- **Local minima vulnerability:** Like all APF methods, the system is theoretically susceptible to local minima in highly symmetric traps. While the specific scenarios in this study were resolved through iterative tuning, a more generalized solution requires integrating the reactive LbCS with a global path planner (e.g., A* or RRT*) to provide geometric escape routes from complex local minima.
- **Heuristic parameter tuning:** Currently, the control parameters and repulsive gains are determined via an iterative, scenario-specific tuning process. To enhance environmental adaptability and reduce reliance on brute-force calibration, future work will integrate principled parameter-synthesis techniques. Specifically, metaheuristic optimization algorithms (such as Particle Swarm Optimization) or adaptive control laws will be explored to dynamically tune the Lyapunov function parameters in real time based on environmental density.

#### 6.1.2 Communication and Scalability.

- **Ideal communication topology:** The model assumes instantaneous, zero-latency state exchange for calculating the swarm centroid and tracking moving obstacles. Real-world networks suffer from latency, packet loss, and signal degradation. Future research will evaluate the controller’s resilience to information delays and develop consensus protocols that function under limited or intermittent bandwidth.
- **Scalability analysis:** While the computational complexity per agent is low, physical swarms face volumetric congestion issues as density increases. Large-scale simulations are required to investigate emergent behaviors and determine the critical density thresholds for stable, collision-free navigation.

#### 6.1.3 Physical Realizability.

- **Simulation-to-reality gap:** The results presented are numerical simulations. Physical deployment introduces unmodeled low-level dynamics, including tire-ground friction (wheel slip), sensor noise, and actuator saturation limits.
- **Hardware validation:** The immediate next step for this research is the transition from simulation to a physical prototype. This involves deploying the acceleration controllers on a fleet of robotic platforms or within a high-fidelity physics engine such as Gazebo to validate the kinematic assumptions and systematically tune the damping gains against real-world environmental constraints.

## 7 Conclusion

This paper addressed the challenge of coordinating a swarm of Segway robots for autonomous navigation in complex and time-varying urban environments. A unified control framework was developed, integrating the intuitive pathfinding of the Artificial Potential Field method with the mathematical rigor of the LbCS. The core of this framework is a novel set of nonlinear, acceleration-based controllers designed to ensure smooth, stable, and predictable motion for the entire multi-agent system.

The primary contributions of this work are fourfold: the design of stabilizing, acceleration-based controllers that mitigate the jerking motion common in velocity-based systems; an enhanced collision avoidance scheme capable of handling inter-agent interactions, static geometric constraints (both disk and line-shaped), and independent dynamic obstacles; the rigorous mathematical proof of system-wide stability, convergence, and asymptotic controllability; and the validation of the framework’s effectiveness through four distinct simulation scenarios. These simulations successfully demonstrated complex, emergent swarm behaviors, including split-and-rejoin maneuvers, formation compression for navigating narrow passages, decentralized rendezvous for a simulated logistics task, and real-time reactive deformation to avoid moving entities.

This research represents a significant step toward creating a reliable and mathematically verifiable control system for autonomous swarms in smart city applications. By providing a robust foundation for managing a fleet of non-holonomic robots in environments with complex geometries and moving entities, this work directly addresses the need for advanced multi-agent coordination. The practical applications of the proposed LbCS framework are extensive, offering direct utility for automated warehouse logistics, coordinated search-and-rescue operations, and the safe, collision-free navigation of autonomous personal mobility vehicles (e.g., smart wheelchairs) in dynamic, human-centric environments. Future work will focus on incorporating predictive estimators to handle highly uncertain dynamic states. To bridge the gap between numerical simulation and real-world deployment, these acceleration-level controllers will undergo rigorous experimental validation on a physical fleet of Segway platforms.

## Supporting information

S1 Appendix Stability and controllability proofs. This file contains the formal mathematical proofs for system convergence using LaSalle’s Invariance Principle and the asymptotic controllability analysis using Sontag’s theorem.
(PDF)
