# Supplementary material for: Maximizing mobility: Distributed controllers of a Segway swarm for autonomous navigation in smart city environments
Source: PLoS One. 2026 Aug 28;21(8):e0356369. doi: 10.1371/journal.pone.0356369 (PMC13524350; doi:10.1371/journal.pone.0356369)
Supplement: Supplementary file 1 [file pone.0356369.s001.pdf]

# Appendix: Stability and controllability proofs

## 1 Convergence analysis

This section provides a formal mathematical proof of the system's convergence using **LaSalle's Invariance Principle** [1]. The domain $D(L(\mathbf{x}))$ constitutes the set of all valid, collision-free states where all physical and operational constraints are satisfied. Mathematically, it is defined as the set where all potential functions appearing in the denominator of the total Lyapunov function $L(\mathbf{x})$ remain strictly positive. Because the state of the augmented system consists of $5n$ variables for the Segway agents and $4p$ variables for the dynamic obstacles, the domain is a subset of $\mathbb{R}^{5n+4p}$:

$$\begin{aligned} D(L(\mathbf{x})) := \Big\{ \mathbf{x} \in \mathbb{R}^{5n+4p} : \; & A_{ij}(\mathbf{x}) > 0, \quad W_{ik}(\mathbf{x}) > 0, \quad B_{im}(\mathbf{x}) > 0, \\ & H_m(\mathbf{x}) > 0, \quad B_{L_i}(\mathbf{x}) > 0, \quad B_{R_i}(\mathbf{x}) > 0, \\ & N_{ih}(\mathbf{x}) > 0, \quad A_{d_{hl}}(\mathbf{x}) > 0, \quad W_{d_{hk}}(\mathbf{x}) > 0, \\ & B_{d_{hm}}(\mathbf{x}) > 0, \quad H_{1_h}(\mathbf{x}) > 0, \quad H_{2_h}(\mathbf{x}) > 0, \\ & \forall i, j \in \{1, \ldots, n\} \text{ with } i \neq j, \\ & \forall h, l \in \{1, \ldots, p\} \text{ with } h \neq l, \\ & \forall k \in \{1, \ldots, q\}, \text{ and } \forall m \in \{1, \ldots, M\} \Big\}. \end{aligned} \tag{1}$$

The following definitions and theorems are utilized to prove stability and convergence.

**Definition 1.1** (Invariant Set)**.** *A set $M \subset D(L(\mathbf{x}))$ is an invariant set of the system if any solution $\mathbf{x}(t)$ initiating from a point in $M$ remains within $M$ for all subsequent time. Formally:*

$$\mathbf{x}(t_0) \in M \implies \mathbf{x}(t) \in M, \quad \forall t \geq t_0.$$

**Theorem 1.2** (LaSalle's Invariance Principle)**.** *Consider a scalar function $L(\mathbf{x})$ defined on $D(L(\mathbf{x}))$ having continuous first-order partial derivatives and satisfying $\dot{L}(\mathbf{x}) \leq 0$. Let $S \subset D(L(\mathbf{x}))$ be the set of all points where $\dot{L}(\mathbf{x}) = 0$. If a solution $\mathbf{x}(t)$ is bounded, then it converges to $M$, the largest invariant set contained in $S$, as $t \to \infty$.*

**Theorem 1.3** (Stability of the Equilibrium Point)**.** *The equilibrium point $\mathbf{x}_e$ of the augmented system is stable in the sense of Lyapunov if the acceleration controllers are defined as in equations (53), (54), (55), and (56).*

*Proof.* The total potential $L(\mathbf{x})$ serves as a valid Lyapunov function satisfying the following conditions:

1. $L(\mathbf{x}) > 0, \quad \forall \mathbf{x} \in D(L(\mathbf{x})), \mathbf{x} \neq \mathbf{x}_e$,
2. $L(\mathbf{x}_e) = 0$,
3. $\dot{L}(\mathbf{x}) = -\sum_{i=1}^{n} (\sigma_{1_i} v_{R_i}^2 + \sigma_{2_i} v_{L_i}^2) - \sum_{h=1}^{p} (\delta_{1_h} v_{d_h}^2 + \delta_{2_h} w_{d_h}^2) \leq 0, \quad \forall \mathbf{x} \in D(L(\mathbf{x}))$.

These conditions confirm that $\mathbf{x}_e$ is a stable equilibrium for the augmented system. □

**Conclusion of convergence:** Theorem 1.3 establishes Lyapunov stability, while Theorem 1.2 guarantees that all bounded solutions converge to the largest invariant subset of $S = \{\mathbf{x} \in D(L(\mathbf{x})) : \dot{L}(\mathbf{x}) = 0\}$. The properties of this invariant set $M$ are analyzed through the following logical steps:

1. **Zero velocity condition:** From the expression of $\dot{L}(\mathbf{x})$, the condition $\dot{L}(\mathbf{x}) = 0$ implies that $-\sum(\sigma_{1_i} v_{R_i}^2 + \sigma_{2_i} v_{L_i}^2) - \sum(\delta_{1_h} v_{d_h}^2 + \delta_{2_h} w_{d_h}^2) = 0$. Since the convergence parameters $\sigma_{1_i}, \sigma_{2_i}, \delta_{1_h}, \delta_{2_h}$ are strictly positive, this equality holds if and only if $v_{R_i} = 0$, $v_{L_i} = 0$, $v_{d_h} = 0$, and $w_{d_h} = 0$ for all swarm agents and dynamic obstacles. Thus, any state in $S$ corresponds to a completely stationary environment.

2. **Zero acceleration condition:** For a state to belong to the *invariant* set $M$, the system must remain in $S$ for $t \to \infty$. The time derivatives of the velocities (accelerations) must also be zero: $a_{R_i} = 0$, $a_{L_i} = 0$, $u_{1_h} = 0$, and $u_{2_h} = 0$.

3. **Zero gradient condition:** Substituting the zero-velocity and zero-acceleration conditions into the derived control laws (equations 53, 54, 55, and 56) reduces the expressions to $\Psi_{R_i} = 0$, $\Psi_{L_i} = 0$, $\frac{\partial L}{\partial x_{d_h}} = 0$, and $\frac{\partial L}{\partial y_{d_h}} = 0$. This implies that the net force (potential gradient) acting on every entity in the workspace is zero: $\nabla L(\mathbf{x}) = 0$.

Therefore, the augmented system asymptotically converges to the set of critical points where the gradient of the potential function vanishes. The target $\mathbf{x}_e$ is the global minimum of $L(\mathbf{x})$ by design. Under the standard assumption that the obstacle configuration does not induce local minima traps, the largest invariant set $M$ is restricted to $M = \{\mathbf{x}_e\}$, guaranteeing that the Segway swarm and all dynamic obstacles successfully converge to their respective target configurations.

## 2 Controllability analysis

The system's asymptotic controllability is formally analyzed using Sontag's theorem, which provides a Lyapunov-like characterization of this property for nonlinear systems [2].

**Theorem 2.1.** *[Sontag, 1983] The system $\dot{\mathbf{x}}(t) = f(\mathbf{x}(t), \mathbf{u}(t))$ is asymptotically controllable if and only if there exists a control Lyapunov function (CLF), $L(\mathbf{x})$, that satisfies the following properties:*

*(a)* $L(\mathbf{x})$ *is continuous.*

*(b)* $L(\mathbf{x})$ *is positive definite, meaning* $L(\mathbf{x}) > 0$ *for all* $\mathbf{x} \neq \mathbf{x}_e$ *and* $L(\mathbf{x}_e) = 0$.

*(c)* $L(\mathbf{x})$ *is proper, meaning the set* $\{\mathbf{x} \mid L(\mathbf{x}) \leq c\}$ *is bounded for any positive constant* $c$.

*(d) For each state* $\mathbf{x} \neq \mathbf{x}_e$*, there exists a control input* $\mathbf{u}$ *such that the time derivative* $\dot{L}(\mathbf{x}) < 0$.

**Theorem 2.2.** *The augmented system defined in (9) is asymptotically controllable if the acceleration controllers are defined as derived in (53), (54), (55), and (56).*

*Proof.* To prove asymptotic controllability, it is demonstrated that the designed total Lyapunov function $L(\mathbf{x})$ satisfies the four CLF conditions stated in Theorem 2.1.

(a) **Continuity of $L(\mathbf{x})$:** The total potential function $L(\mathbf{x})$ is composed of target attraction terms $(T(\mathbf{x}), V_h(\mathbf{x}))$, auxiliary functions $(F(\mathbf{x}), G_{d_h}(\mathbf{x}))$, and various barrier functions representing constraints for the swarm $(A_{ij}, W_{ik}, B_{im}, H_m, B_{R_i}, B_{L_i})$ and the dynamic obstacles $(N_{ih}, A_{d_{hl}}, W_{d_{hk}}, B_{d_{hm}}, H_{1_h}, H_{2_h})$. All constituent functions are polynomials or

rational functions that are continuous and differentiable strictly within the domain $D(L(\mathbf{x}))$. Because the domain is defined as the open set where all denominator terms remain strictly positive, $L(\mathbf{x})$ is continuous on $D(L(\mathbf{x}))$.

(b) **Positive definiteness:** As established in the convergence analysis, $L(\mathbf{x})$ is strictly positive for all $\mathbf{x} \in D(L(\mathbf{x}))$ where $\mathbf{x} \neq \mathbf{x}_e$, and vanishes only at the unique global equilibrium $\mathbf{x}_e$. Thus, $L(\mathbf{x})$ is positive definite.

(c) **Properness:** A function is proper if its sublevel sets are compact. This ensures that the system cannot escape to infinity or violate an obstacle boundary while maintaining finite energy. Structurally, as any agent or dynamic obstacle approaches a collision state or a velocity limit (the boundary of $D(L(\mathbf{x}))$), the denominator terms in the respective barrier functions vanish, causing the potential value $L(\mathbf{x})$ to approach infinity. Similarly, as any entity moves infinitely far from its target ($\|\mathbf{x}\| \to \infty$), the quadratic attraction terms $T(\mathbf{x})$ and $V_h(\mathbf{x})$ cause $L(\mathbf{x}) \to \infty$. Because the potential field boundaries are infinitely high, any trajectory initiating with finite energy $L(\mathbf{x}_0) = \eta$ is strictly confined within the compact set $\{\mathbf{x} \mid L(\mathbf{x}) \leq \eta\}$.

(d) **Existence of stabilizing control:** It must be shown that for any $\mathbf{x} \neq \mathbf{x}_e$, there exists a control vector $\mathbf{u}$ such that $\dot{L}(\mathbf{x}) < 0$.

The derived control laws yield the time derivative:

$$\dot{L}(\mathbf{x}) = -\sum_{i=1}^{n}(\sigma_{1_i} v_{R_i}^2 + \sigma_{2_i} v_{L_i}^2) - \sum_{h=1}^{p}(\delta_{1_h} v_{d_h}^2 + \delta_{2_h} w_{d_h}^2) \leq 0.$$

This derivative is strictly negative if any Segway wheel or dynamic obstacle is in motion. The critical case occurs when the entire augmented system is momentarily stationary ($\mathbf{v} = \mathbf{0}$) at a configuration $\mathbf{x} \neq \mathbf{x}_e$.

At such a state, because $\mathbf{x} \neq \mathbf{x}_e$, the gradient of the potential function is non-zero ($\nabla L(\mathbf{x}) \neq 0$). The acceleration controllers are proportional to this gradient. For the Segways, $a \propto \Psi$, and for the dynamic obstacles, $u \propto \nabla L$. Consequently, the controllers generate a non-zero acceleration vector $\mathbf{u} \neq \mathbf{0}$. This acceleration forces the system to immediately exit the zero-velocity manifold, thereby inducing motion and ensuring $\dot{L}(\mathbf{x}) < 0$ in the subsequent instant.

Furthermore, as $\mathbf{x} \to \mathbf{x}_e$, the velocity components and the spatial gradient terms vanish smoothly, ensuring the total control input $\mathbf{u}(\mathbf{x})$ remains bounded and continuous throughout the operational space.

Thus, the augmented system is asymptotically controllable. □

# References


1. LaSalle J. Some extensions of Liapunov's second method. IRE Transactions on circuit theory. 1960;7(4):520–527.

2. Sontag ED. A Lyapunov-like characterization of asymptotic controllability. SIAM journal on control and optimization. 1983;21(3):462–471.
